# Bi-allelic variants in human *WDR63* cause male infertility via abnormal inner dynein arms assembly

**DOI:** 10.1038/s41421-021-00327-5

**Published:** 2021-11-16

**Authors:** Shuai Lu, Yayun Gu, Yifei Wu, Shenmin Yang, Chenmeijie Li, Lanlan Meng, Wenwen Yuan, Tao Jiang, Xin Zhang, Yang Li, Cheng Wang, Mingxi Liu, Lan Ye, Xuejiang Guo, Hongbing Shen, Xiaoyu Yang, Yueqiu Tan, Zhibin Hu

**Affiliations:** 1 Department of Epidemiology, School of Public Health, Southeast University, Nanjing, Jiangsu China; 2 State Key Laboratory of Reproductive Medicine, Nanjing Medical University, Nanjing, Jiangsu China; 3 Department of Epidemiology, Center for Global Health, School of Public Health, Nanjing Medical University, Nanjing, Jiangsu China; 4 State Key Laboratory of Reproductive Medicine, The Affiliated Suzhou Hospital of Nanjing Medical University, Suzhou, Jiangsu China; 5 Suzhou Municipal Hospital, Suzhou, Jiangsu China; 6 Institute of Reproductive and Stem Cell Engineering, School of Basic Medical Science, Central South University, Changsha, Hunan China; 7 Clinical Research Center for Reproduction and Genetics in Hunan Province, Reproductive and Genetic Hospital of CITIC-Xiangya, Changsha, Hunan China; 8 Center of Reproductive Medicine, First Affiliated Hospital of Nanjing Medical University, Nanjing, Jiangsu China

**Keywords:** Developmental biology, Mechanisms of disease

## Abstract

Inner dynein arm (IDA), composed of a series of protein complex, is necessary to cilia and flagella bend formation and beating. Previous studies indicated that defects of IDA protein complex result in multiple morphological abnormalities of the sperm flagellum (MMAF) and male infertility. However, the genetic causes and molecular mechanisms in the IDAs need further exploration. Here we identified two loss-of-function variants of *WDR63* in both MMAF and non-obstructive azoospermia (NOA) affected cohorts. *WDR63* encodes an IDA-associated protein that is dominantly expressed in testis. We next generated *Wdr63*-knockout (*Wdr63*-KO) mice through the CRISPR-Cas9 technology. Remarkably, *Wdr63*-KO induced decreased sperm number, abnormal flagellar morphology and male infertility. In addition, transmission electron microscopy assay showed severely disorganized “9 + 2” axoneme and absent inner dynein arms in the spermatozoa from *Wdr63*-KO male mice. Mechanistically, we found that WDR63 interacted with WDR78 mainly via WD40-repeat domain and is necessary for IDA assembly. Furthermore, WDR63-associated male infertility in human and mice could be overcome by intracytoplasmic sperm injection (ICSI) treatment. In conclusion, the present study demonstrates that bi-allelic variants of WDR63 cause male infertility via abnormal inner dynein arms assembly and flagella formation and can be used as a genetic diagnostic indicator for infertility males.

## Introduction

Infertility is a global human health concern and affects approximately 8%–12% of couples around the world^1,2^. Male infertility, which accounts for half of all infertility cases, is generally manifested by reduced sperm count (oligozoospermia and azoospermia), reduced sperm motility (asthenozoospermia), or increased percentage of abnormal sperm morphology (teratozoospermia). Combinations are common, such as “asthenoteratozoospermia” and “oligoasthenoteratozoospermia or OAT syndrome”^3,4^. The flagellum integrity is essential for normal sperm function and flagellum defects consistently result in male infertility^5^. Multiple morphological abnormalities of the sperm flagellum (MMAF) is one of the most severe asthenoteratozoospermia, which is usually combined with reduced sperm count and is characterized by abnormal flagellar formation, including absent, short, coiled, bent flagellar, and/or irregular caliber^6,7^.

Genetic factors account for at least 15% of male infertility, and MMAF is currently one of the most studied male infertility disease with genetic factors^8,9^. With the wide application of high-throughput sequencing technologies, such as whole-exome sequencing (WES), a series of MMAF-associated genes have been identified through genetic studies and animal models. These studies are relevant for MMAF diagnostic value, clinical decision making, and appropriate genetic counseling^10–13^. Owing to high genetic heterogeneity, only approximately 60% of MMAF-affected cases are caused by previously identified genetic factors, indicating the necessary to discover new potential MMAF-associated genes.

Motile cilia or flagella are evolutionarily conserved from unicellular protozoa to mammals^14^. Sperm flagella typically comprise a “9 + 2” axonemal arrangement and a number of multi-protein complexes, including outer dense fiber (ODF), fibrous sheath (FS), nexin-dynein regulatory complex (N-DRC), calmodulin-and spoke-associated complex(CSC) and dynein arms(DAs)^15^. Among these protein complexes, the inner dynein arms (IDAs) composed of two heavy chains 1αHC (DNAH10, in mammals, similarly hereinafter) and 1β HC (DNAH2), three intermediate chains, IC140 (WDR63), IC138 (WDR78) and IC97 (LAS1) and several light chains in *Chlamydomonas*^14,16^. This subcomplex is absolutely required for flagella integrity and assembly, and is responsible for cilia (or flagella) beat frequency and bend formation^16^. For example, functional defective for the WDR78 are completely unable to assemble these structures into the axoneme in vertebrate^17^. Previous studies have shown that variants in IDAs protein complex result in several ciliopathies and male infertility. In particular, bi-allelic in *DNAH1* (MIM: 603332), an IDA-associated gene, contributes to MMAF syndrome in human and mice^18^. However, the genetic causes and molecular mechanisms in the other components of IDAs need further exploration.

Here, we identified two bi-allelic variants of *WDR63* in both MMAF and NOA-affected cohorts. Furthermore, WDR63 forms a complex with the IDA component WDR78, and these two proteins co-localized in the sperm flagella. *Wdr63*-KO impaired the assembly of IDA and caused severely decreased sperm number, sperm motility and abnormal flagellar morphology. Remarkably, intracytoplasmic sperm injection (ICSI) treatment revealed that both *Wdr63*-KO mice and men harboring *WDR63* variants acquired successful clinical pregnancy. Our study suggests that bi-allelic variants of *WDR63* can be used as an inherited pathogenic factor and a genetic diagnostic indicator for infertility males.

## Results

### Identification of bi-allelic variants of *WDR63* in infertility men

**Fig. 1:**
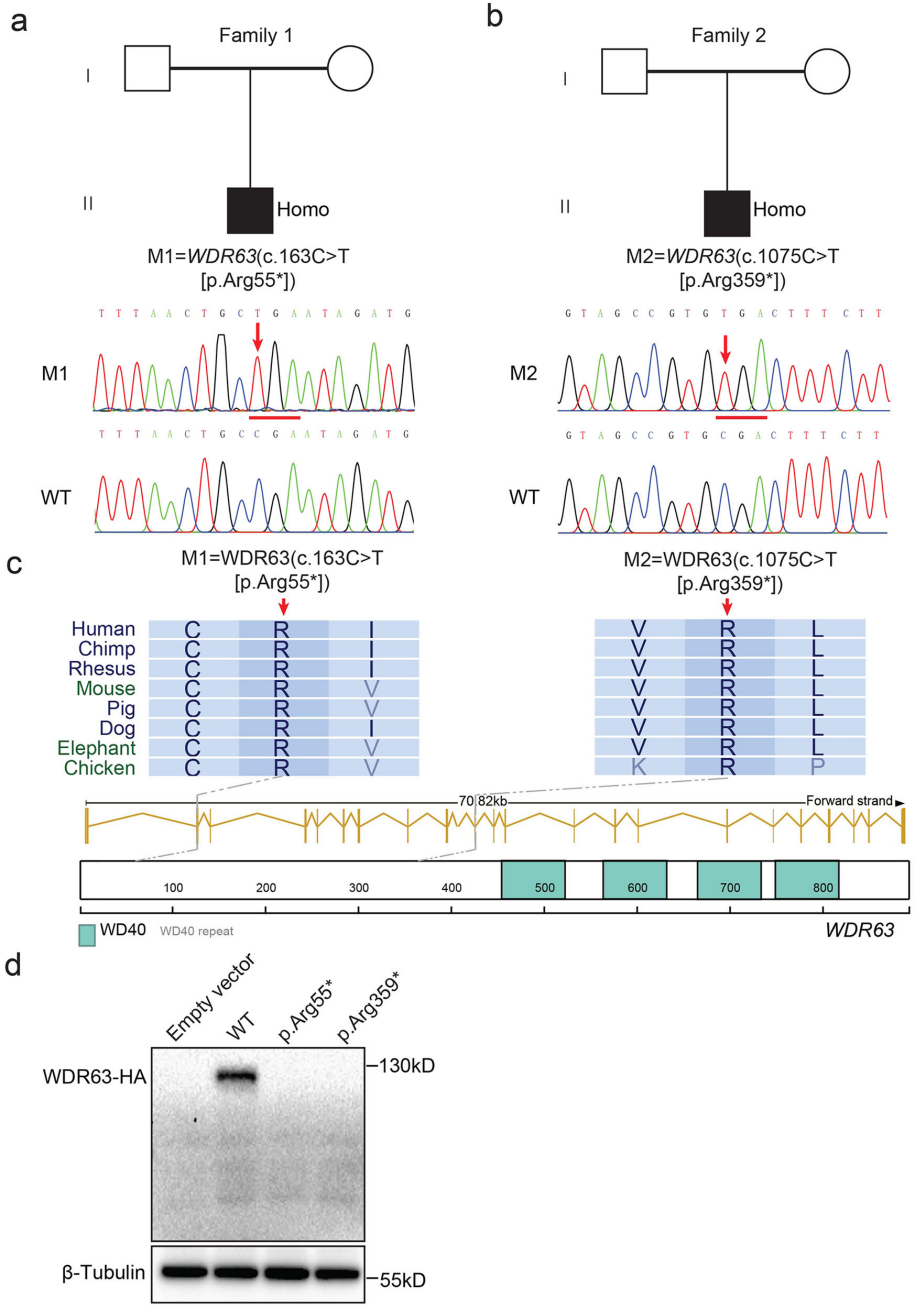
Identification of bi-allelic variants of *WDR63* in patients with MMAF. **a**, **b** Pedigree of family 1 affected by bi-allelic *WDR63* variant that was identified by WES from MMAF-affected men (a). Pedigree of family 2 affected by bi-allelic *WDR63* variant that were identified by Sanger sequencing from NOA-affected men (b). Filled black filled squares indicate infertile men in this family. Sanger sequencing results are shown under the pedigrees. The mutated positions are indicated by red arrows. **c** Schematic representation of the domains of WDR63 and locations of *WDR63* variants identified in this study. Sequence alignment shows conservation of the mutated residues across different species according to UCSC genome browser. The green boxes indicate Trp-Asp (WD40) repeat domains as described by the UniProt server. **d** *WDR63* variants cause the degradation of WDR63 protein. Full-length wild-type and mutant *WDR63* cDNA constructs were overexpressed in HEK293T cells followed by immunoblotting analysis. Abbreviations: M1, mutation 1; M2, mutation 2; Homo, homozygous; WD40, Trp-Asp repeat.

To identify the potential IDA-associated variants in male infertility, we conducted whole-exome sequencing (WES) analyses in a distinct MMAF cohort depend on variants frequency and functional annotation (minor allele frequency [MAF] < 0.001 in the gnomAD database and combined annotation-dependent depletion [CADD] score of ≥ 15) (Supplementary Fig. S1). In the cohort of 243 MMAF-affected Chinese men, we identified a harboring homozygous stop-gain variant (M1: c.163 C > T [p.Arg55*], CADD = 37) in *WDR63* (MIM: 617968; NCBI: NM_145172.5) (Fig. 1a and Table 1). Semen parameters of men harboring bi-allelic *WDR63* variant was analyzed in the source laboratories according to WHO guidelines^19^. The typical MMAF characteristic was observed in the spermatozoa from the men harboring bi-allelic *WDR63* variant. Notably, the sperm counts were dramatically lower than the normal reference values (Supplementary Table S1). In addition, we identified an additional bi-allelic variant of *WDR63* in 121 non-obstructive azoospermia (NOA) male (M2: c.1075 C > T [p.Arg359*], CADD = 35) using Sanger sequencing (Fig. 1b and Table 1), indicating that variants of *WDR63* might cause the severely decrease in sperm counts.

**Table 1:** Bi-allelic *WDR63* variants identified in infertility males.

	M1	M2
cDNA alteration	c.163 C > T	c.1075 C > T
Variant allele	Homozygous	Homozygous
Protein alteration	p.Arg55*	p.Arg359*
Variant type	Nonsense	Nonsense
Allele frequency in human population		
ExAC(all/Asian)	3.3 × 10^−05^/0.8 × 10^−04^	1.3 × 10^−04^/0
gnomAD (all/Asian)	1.3 × 10^−05^/0.4 × 10^−04^	2.9 × 10^−04^/0
Function prediction		
SIFT	Damaging	Damaging
PolyPhen-2	Damaging	Damaging
Mutation Taster	Damaging	Damaging
CADD	37	35

NCBI reference sequence number of WDR63 is NP_660155.2.

Human *WDR63* contains 23 exons and encodes a predicted 891-amino-acid protein that comprises four Trp-Asp (WD40) repeat domains (NCBI: NP_660155.2; UniProt: Q8IWG1). In this study, all of variants in *WDR63* are located before the WD40 domain, leading to the loss of WD40 domains in WDR63 protein (Fig. 1c). We overexpressed full-length wild-type and mutant cDNA constructs in HEK293T cells and found the significantly absence of mutant cDNA constructs of WDR63 (Fig. 1d). Based on above results, we speculate that the bi-allelic loss-of-function (LOF) variants of *WDR63* is one of the genetic causes of male infertility.

### WDR63 is indispensable for male fertility in mice

**Fig. 2:**
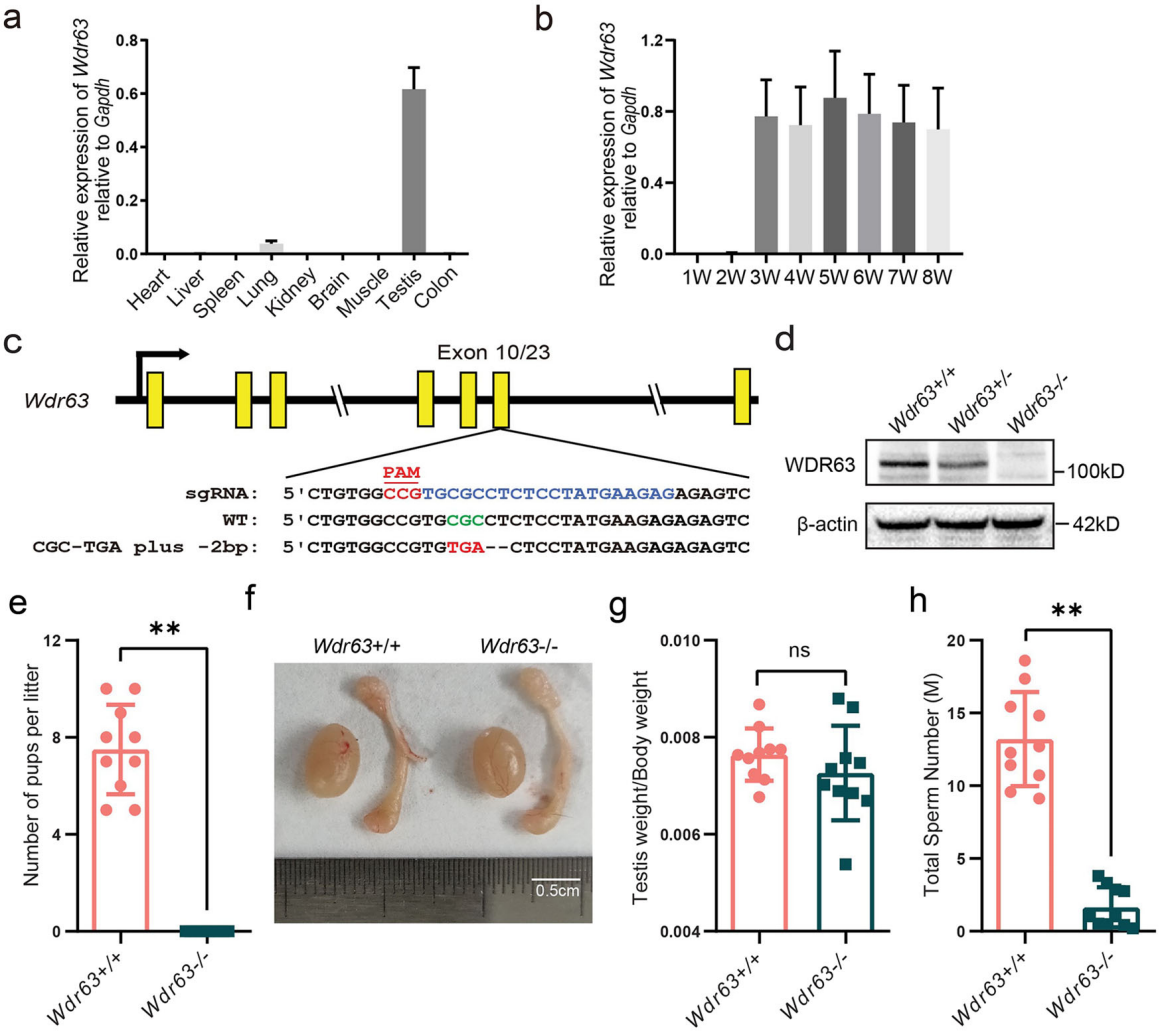
WDR63 protein is highly expressed in late stages of spermatogenesis and *Wdr63*-null males are infertile. **a** Real-time q-PCR for *Wdr63* transcripts in various 8-weeks mouse tissues, and *Gapdh* gene is used as a control. Error bars, SEM (*n* = 3). **b** mRNA levels of *Wdr63* in mouse testis at the indicated time points. Error bars, SEM (*n* = 3). **c** Schematic diagram of CRISPR/Cas9 strategy for the generation of *Wdr63* KO mice. The sgRNA was designed to target exon 10 of the *Wdr63*, a nonsense mutation (GCG-TGA) plus a 2-bp deletion was obtained. **d** Western blot analysis of WDR63 protein in wild-type (*Wdr63*^+/+^), heterozygous (*Wdr63*^*+/−*^) and homozygous (*Wdr63*^*−/−*^) mice with a mouse polyclonal antibody raised against WDR63. β-actin was used as a loading control. **e** Fertility analysis for adult *Wdr63*^+/+^ and *Wdr63*^−/−^. **f**, **g** The morphology and testis weight of testes and epididymis from *Wdr63*^+/+^ and *Wdr63*^*−/−*^ males at 8-week-old. Scale bars, 0.5 cm. **h** Total sperm number was dramatically decreased in *Wdr63*^*−/−*^ males compared to that of *Wdr63*^+/+^ males. Abbreviations: W, week; M, million. For **e** to **h**, *n* = 10 and the bars represent means ± SEM. The statistical analysis was carried out using One-way ANOVA test, ** denotes *P* < 0.01; ns, not significant.

To explore the biological function of WDR63 during spermatogenesis, we first characterized tissue-specific distribution of *Wdr63* mRNA transcript. Quantitative RT-PCR (q-PCR) analysis revealed that *Wdr63* transcript was preferentially expressed in testis tissue both in human and mice (Fig. 2a and Supplementary Fig. S2a). We further determined *Wdr63* transcript levels in mouse testis at different developmental stages. *Wdr63* transcript was absent in mouse testis at postnatal day 7 (P7) and P14 (Fig. 2b), when germ cells differentiate to spermatogonia and pachytene spermatocytes, respectively. Further analysis revealed that *Wdr63* was significantly increased in mouse testis at P21, when round spermatids appear (Fig. 2b).

Considering that mouse WDR63 is highly homologous to human WDR63 protein and primarily expressed in mice testes (Supplementary Fig. S2b), we generated *Wdr63*-KO mice using CRISPR-Cas9 technology. Sanger sequencing of *Wdr63* homozygous mutated mice confirmed the presence of a nonsense mutation plus a 2-bp deletion in exon 10, which was predicted to cause premature translational termination (p.Arg362*) (Fig. 2c). Compared with wild-type (WT) male mice, immunoblotting indicated that WDR63 was absent and reduced in homozygous and heterozygous male mice testes, respectively (Fig. 2d). To test male fertility, individual males (wild-type and homozygous) were housed with wild-type females for 2 months. The number of offspring per litter was recorded. Our data revealed that *Wdr63*-KO male mice were completely infertile, although they were sexually active and produced vaginal plugs in female partners (Fig. 2e). We also compared testis weights and sizes between WT and *Wdr63*-KO male mice, but no significant differences were found (Fig. 2f, g). In addition, the same phenotypes were found in *Wdr63*-null male mice with 7-bp frameshift deletion (Supplementary Fig. S3a–j). Consistent with human mutation individuals, these experimental observations indicate that WDR63 is necessary for male fertility in mice.

### *Wdr63*-KO male mice showed severe oligozoospermia combined with MMAF phenotypes

**Fig. 3:**
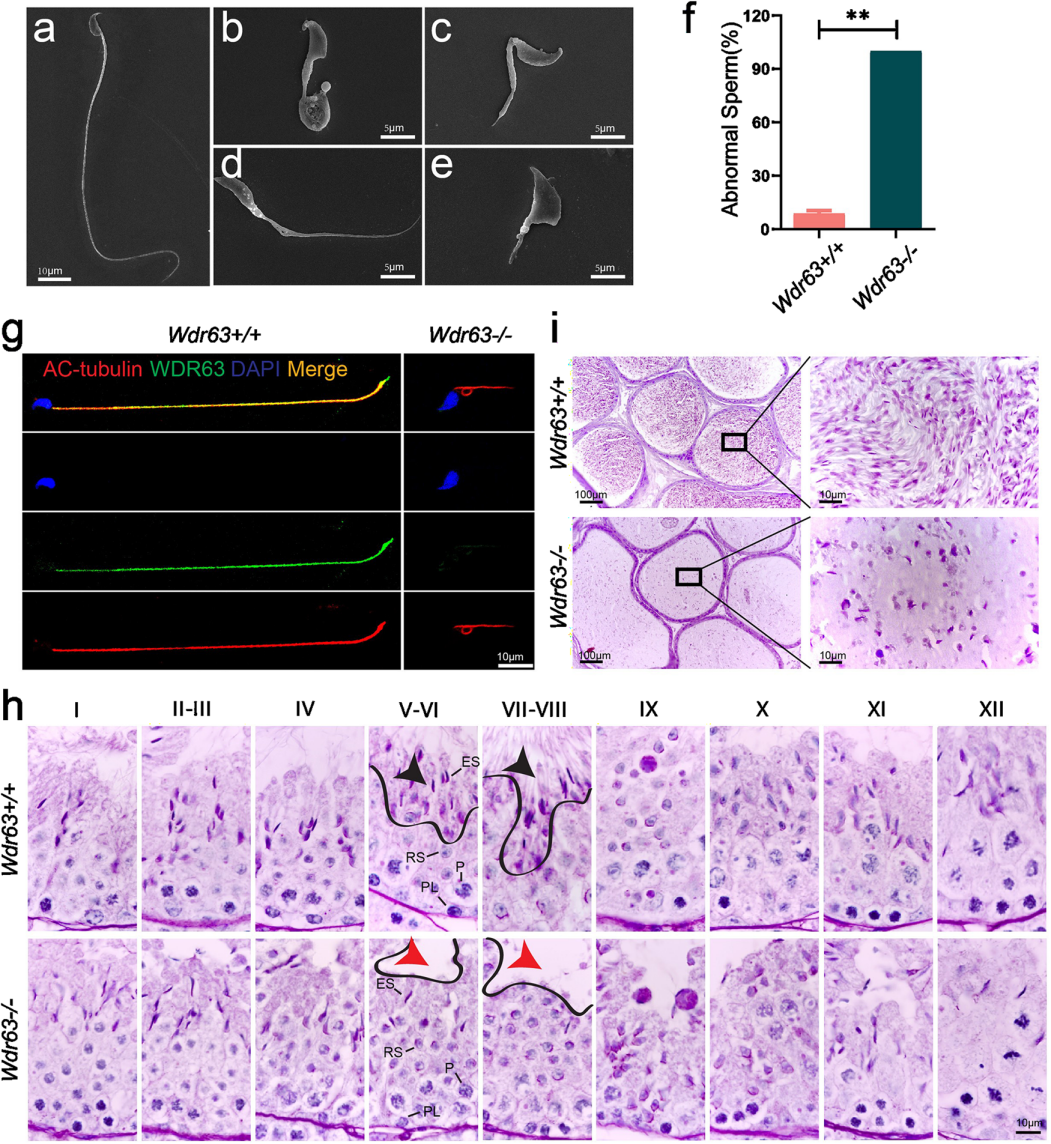
*Wdr63*-KO males showed typical MMAF phenotypes. **a** SEM shows a spermatozoon with normal morphology from the epididymis of *Wdr63*^+/+^ males. Scale bars, 10 μm. **b**–**e** SEM shows the spermatozoon of *Wdr63*^*−/−*^ males presented with MMAF phenotypes, such as short and coiled flagella, flagella of irregular caliber, and other malformations diagnosed as MMAF. Scale bars, 5 μm. **f** Spermatocytograms showing the number of abnormal sperms in WT and homozygous mutant males. **g** Immunofluorescent staining of WDR63 (green), AC-Tubulin (red) and DAPI (blue) on the spermatozoa collected from *Wdr63*^+/+^ and *Wdr63*^*−/−*^ males. WDR63 localized at entire flagella in WT male mice. Scale bars, 10 μm. **h** Periodic acid–Schiff (PAS) staining of testis sections from adult *Wdr63*^+/+^ and *Wdr63*^*−/−*^ mice. Black arrows indicated normal spermatozoon flagella in stage VII–VIII seminiferous tubules of *Wdr63*^+/+^ male mice, whereas red arrows indicated the absence of elongated tails in *Wdr63*^*−/−*^ male mice. Scale bars, 10 μm. **i** HE staining of the cauda epididymis from male mice. Decreased sperm quantity was observed in the epididymis from *Wdr63*^*−/−*^ male mice, when compared with that of *Wdr63*^*+/+*^ male mice. Scale bars, 10 μm. Abbreviations: PL, preleptotene; P, pachytene; RS, round spermatids; ES, elongated spermatids. For **f**, *n* = 3 and the bars represent means ± SEM. The statistical analysis was carried out using one-way ANOVA test, ** denotes *P* < 0.01.

Having demonstrated the function of WDR63 in male fertility, we next determine the semen characteristics and sperm morphology in *Wdr63*-KO males. We examined sperm function using the Computer Assisted Sperm Analyzer (CASA) method. Compared with WT male mice, no sperm or very few sperm were detected in the epididymis of homozygotic male mice (Fig. 2h). In addition, the sperm motility and sperm progressive rate was as low as zero in *Wdr63*-KO male mice (Table 2 and Supplementary Movies S1 and S2). Consistent with the clinical phenotypes of MMAF-affected men with bi-allelic *WDR63* variants, the sperm flagella of *Wdr63*-KO male mice also presented with absent, short, coiled, bent, and/or irregular shapes (Fig. 3a–f and Table 2). Immunofluorescence assays were performed with the cauda epididymis sperm cells from WT and *Wdr63*-KO male mice. Immunostaining analysis showed that WDR63 co-localized with Acetyl-α-Tubulin (AC-tubulin, a marker of axoneme) along the entire flagella in WT male mice but was almost absent in *Wdr63*-KO male mice, indicating that WDR63 is an essential component of axoneme (Fig. 3g).

**Table 2:** Sperm characteristics and flagellar morphology of Wdr63-KO male mice.

	WT	KO
Semen parameter		
Motility (%)	77.79 ± 8.24	4.83 ± 3.38**
Progressive (%)	40.66 ± 10.30	0.71 ± 0.99**
Sperm flagellar morphology^a^		
Absent flagella (%)	4.05 ± 1.48	21.5 ± 1.49*
Short flagella (%)	0 ± 0	25.84 ± 5.59**
Coiled flagella (%)	0 ± 0	29.12 ± 5.72**
Irregular caliber (%)	1.53 ± 0.63	17.03 ± 1.86**
Bent flagella (%)	3.15 ± 1.09	6.51 ± 1.02

^a^Data represent the means ± SD of three independent experiments.

*P < 0.05, **P < 0.01.

To investigate the role of WDR63 in different stages of spermatogenesis, we performed Periodic acid–Schiff (PAS) staining on the testes of WT and *Wdr63*-KO male mice. Gross examination of testes revealed no difference in spermatogonia, spermatocyte and round spermatids between homozygous and wild-type (Fig. 3h). We next counted the number of spermatids and Sertoli cells in stage VII–VIII seminiferous tubules of male mice testis (Supplementary Fig. S4a). Statistical analysis showed no significant difference in Sertoli cells, spermatogonia, spermatocyte and round spermatids between homozygous and wild-type, but the number of elongated spermatids was dramatically decreased in *Wdr63-*KO mouse (Supplementary Fig. S4b). Besides, the ratios of elongated spermatid and Sertoli cells were decreased in homozygous (0.21 ± 0.18) compared to wild-type (3.24 ± 0.98) (Supplementary Fig. S4c). Hematoxylin and eosin (HE) staining of the cauda epididymis from *Wdr63*-KO male mice displayed fewer sperm heads than those from WT mice (Fig. 3i).

**Fig. 4:**
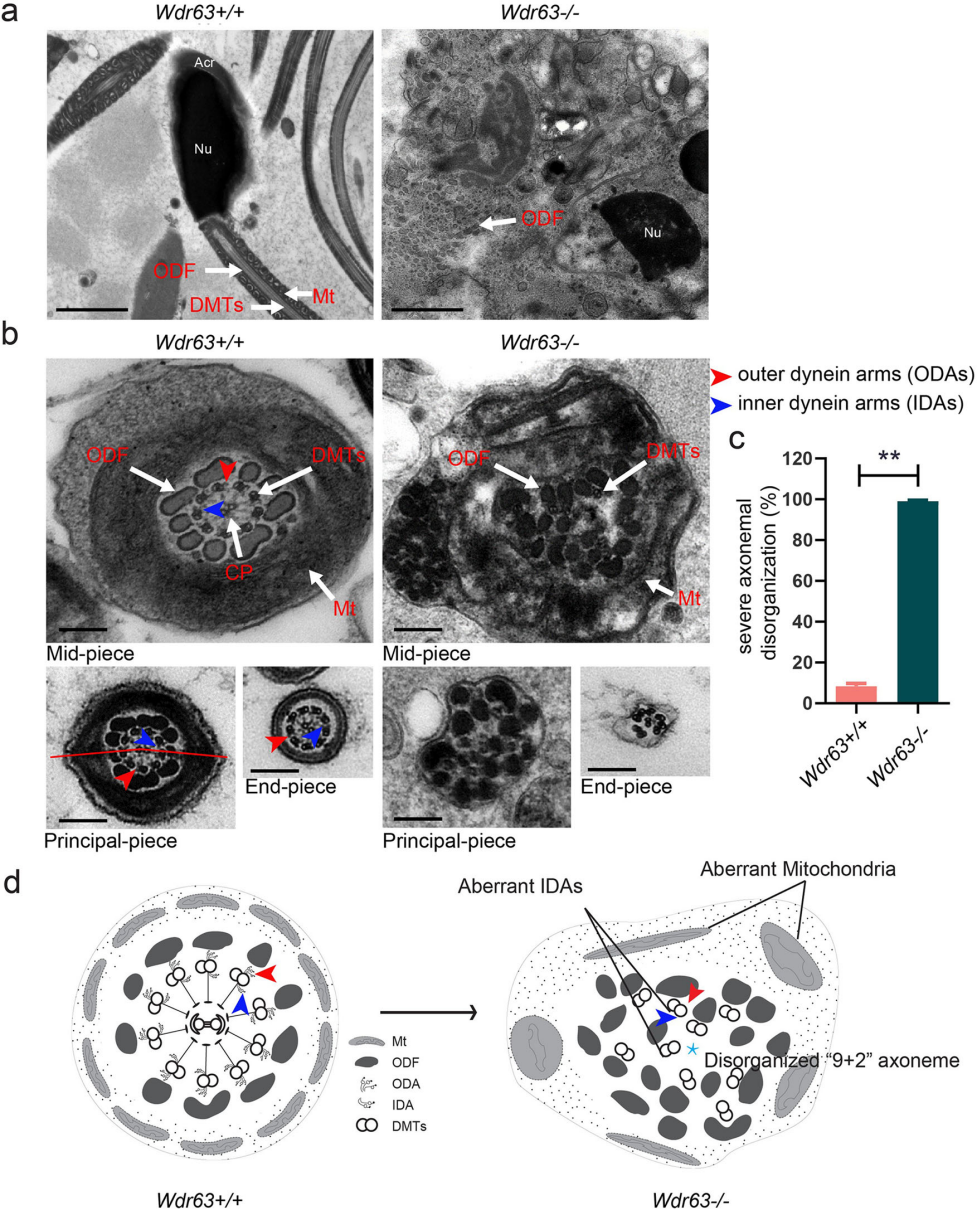
Ultrastructure analyses for *Wdr63*-KO male mice show a severely disorganized axoneme disorganization arrangement. **a** Longitudinal sections of spermatozoon mid-piece in adult male mice. *Wdr63*^*+/+*^ male mice sperm had a symmetrical mid-piece with smooth axoneme surrounding with a regularly arranged mitochondrial sheath. In contrast, *Wdr63*^*−/−*^ male mice showed a seriously disorganized ODF and deficient of axoneme and mitochondrial sheath. Scale bars, 2 μm. **b**, **c** Cross-sections of spermatozoon flagella the mid-piece, principal piece, and end-piece in adult male mice. *Wdr63*^+/+^ male mice show the typical “9 + 2” microtubule structure, whereas nearly all spermatozoon of *Wdr63*^*−/−*^ male mice show severe disorganized “9 + 2” axoneme. Scale bars, 200 nm. **d** Schematic of spermatozoon flagella cross-sections ultrastructure in *Wdr63*^+/+^ and *Wdr63*^*−/−*^ male mice. Abbreviations: Nu, nucleus; Acr, acrosome; DMT, peripheral microtubule doublets; ODF, outer dense fibers; CP, central pair of microtubules; Mt, mitochondrial sheath; ODA, outer dynein arms (red arrows); IDA, inner dynein arms (blue arrows). For **c**, *n* = 3 and the bars represent means ± SEM. The statistical analysis was carried out using One-way ANOVA test, ** denotes *P* < 0.01.

To further investigate the roles of WDR63 in ultra-structures of spermatozoa in cauda epididymis, we conducted Transmission Electron Microscope (TEM) in both WT and *Wdr63*-KO male mice. Scattered and disorganized axonemal components and cytoplasm residue were observed in the sperm necks and flagella of *Wdr63*-KO male mice (Fig. 4a). As show in Fig. 4b, c, cross-sections showed severe disorganized “9 + 2” axoneme combined aberrant inner dynein arm (IDA), outer dynein arm (ODA), outer dense fiber (ODF), fibrous sheath (FS) and nexin-dynein regulatory complex (N-DRC) in the spermatozoa from *Wdr63*-KO male mice. These data indicate that WDR63 is required for sperm flagellar formation (Fig. 4d). In summary, we observe WDR63 deficiency caused decreased sperm counts and typical MMAF phenotypes.

### WDR63 interacts with WDR78 and their association is necessary for IDA assembly

**Fig. 5:**
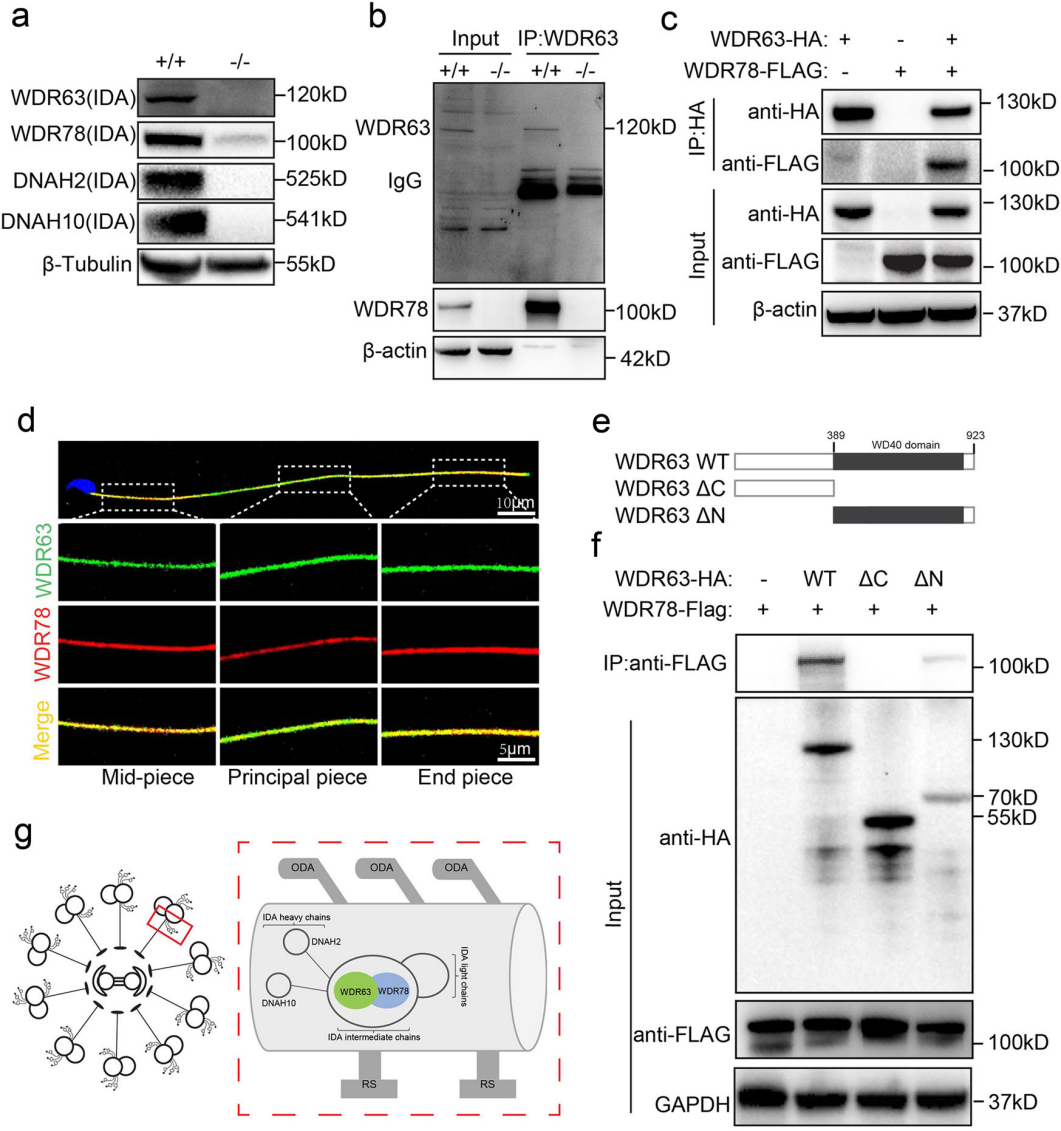
WDR63 associates with WDR78 and their interaction is necessary for IDA assembly. **a** Western blot analysis of WDR63, WDR78, DNAH2 and DNAH10 in testes of *Wdr63*^+/+^ and *Wdr63*^*−/−*^ male mice. **b** Immunoprecipitation with testicular extracts using anti-WDR63 antibodies, Mass spectrometric analysis identified WDR78 is the potential associated proteins. Western blot analysis and confirmed that WDR63 interacts with WDR78. **c** HA-WDR63 and/or FLAG-WDR78 expression constructs were transfected into HEK293T cells. After 48 h, the cells were collected for immunoprecipitation analysis with indicated anti-HA and anti-FLAG antibodies. **d** Immunofluorescence analysis indicated that WDR63 co-localized with WDR78 in normal spermatozoon. **e** Schematic diagram of mouse wild type WDR63 (NP_766452.2) and truncated constructs. WD40 repeats domains are marked in a gray box (398–785 aa, UniProt ID: B2RY71). WDR63 ΔC, deletion of the C terminal domain of WDR63 (398–923 aa); WDR63 ΔN, deletion of the N terminal domain of WDR63 (1–397 aa). **f** HA-WDR63 WT, HA-WDR63 ΔC and HA-WDR63 ΔN were co-transfected with FLAG-WDR78 into HEK293T cells, respectively. After 48 h, the cells were collected for immunoprecipitation analysis with the indicated antibodies. **g** Schematic of the role of WDR63 interacts with WDR78 in IDAs structure.

WDR63 is the vertebrate orthologue of IC140, a subunit of IDA intermediate chains, is critical for assembly of the IDA complex in *Chlamydomonas*^16^. Mammalian IDA is a complex structure attached to the peripheral microtubules through docking complexes^20^, and it is essential for bend formation and beating form of cilia and flagella^21^. To characterize the function of WDR63 in vertebrate flagella formation, we first examined whether the protein levels of the other three IDA subunits, DNAH2, DNAH10 and WDR78 were altered in *Wdr63*-KO testes. Immunoblotting analysis revealed that WDR78 was dramatically decreased, and DNAH2 and DNAH10 were absent in *Wdr63*-KO. This result indicates that WDR63 is essential for maintaining other components of IDA structure and IDA assembly (Fig. 5a). We next detected the mRNA expression levels of *Wdr63*, *Wdr78*, *Dnah2* and *Dnah10* in *Wdr63*-KO and WT mice. q-PCR analysis revealed that the mRNA expression levels of *Wdr63* was decreased, but there was no difference in *Wdr78*, *Dnah2* and *Dnah10* (Supplementary Fig. S5a). To identify protein partners of WDR63, we performed immunoprecipitation with testicular extracts using anti-WDR63 antibodies. Mass spectrometric analysis revealed that WDR78 is the only potential associated protein (Supplementary Data S1). In addition, *Wdr78* transcript was preferentially expressed in testis, and was significantly increased in mouse testis at P21 (Supplementary Fig. S5b, c). Furthermore, the interaction of WDR63 with WDR78 was verified by coimmunoprecipitation (co-IP) assays (Fig. 5b, c). Immunofluorescence analysis also found that WDR63 co-localization with WDR78, further indicating co-expression between WDR63 and WDR78 (Fig. 5d). Besides, we examined whether WDR63 binds to DNAH2 and DNAH10, which are cooperators of WDR63 in *Chlamydomonas*^16^. However, immunoblotting followed by coimmunoprecipitation (co-IP) assays demonstrated that WDR63 did not bind to DNAH2 and DNAH10 in mammals (Supplementary Fig. S5d).

To identify the domain required for the interaction of WDR63 with WDR78, we generated HA-tagged full-length WDR63 protein and tagged truncation constructs lacking either the N terminus (WDR63 ΔN) or the WD40 repeats domain at C terminus (WDR63 ΔC) (Fig. 5e). co-IP assays revealed that the WDR63-WDR78 interaction was disrupted upon the deletion of the WDR63 C terminus, indicating that the WD40 repeats domain at the C terminus mediates their interaction (Fig. 5f). These results suggest that WDR63 forms a complex with WDR78 and the assembly of IDA structure requires WDR63 as an essential subunit (Fig. 5g).

### WDR63-associated male infertility could be rescued by ICSI

**Fig. 6:**
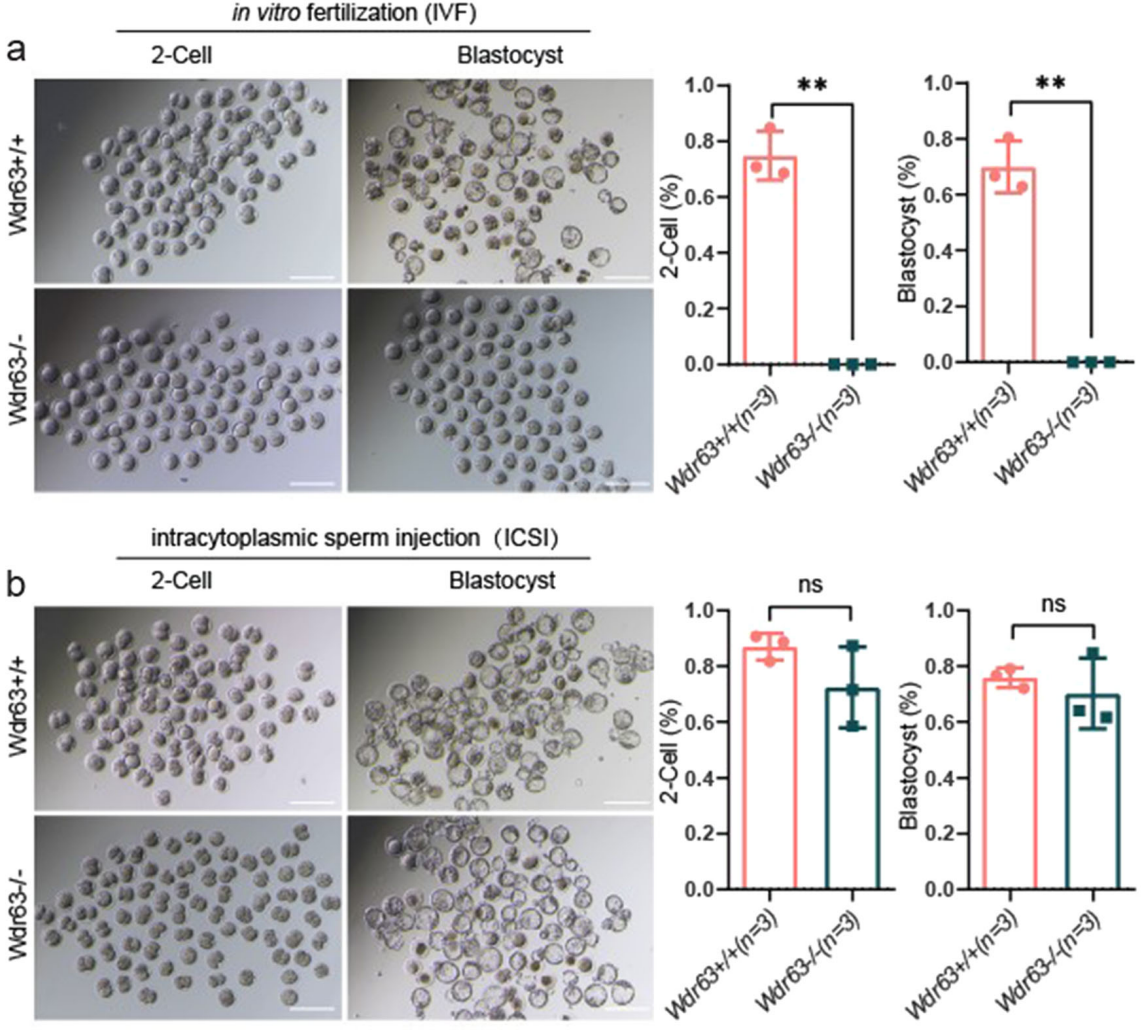
WDR63-associated male infertility could be rescue by ICSI but not IVF. **a** Representative two-cell embryos and blastocysts from in vitro fertilization. Scale bar, 200 μm. Compared with WT male mice, the rates of 2-cell and blastocyst as lower as zero in *Wdr63* KO male mice. **b** Representative two-cell embryos and blastocysts from intracytoplasmic sperm injection. The 2-cell and blastocyst were successfully obtained upon ICSI using the spermatozoa from *Wdr63* KO male mice. Scale bar, 200 μm. For **a** and **b**, *n* = 3 and the bars represent means ± SEM. The statistical analysis was carried out using One-way ANOVA test, ** denotes *P* < 0.01; ns, not significant.

Finally, we aimed to evaluate whether the male infertility induced by WDR63 variants could be overcome by Assisted Reproductive Technology (ART). First, in vitro fertilization (IVF) was utilized to the sperm from WT and *Wdr63*-KO male mice. The two-cell (2-cell) rates and blastocyst rates were recorded after caudal epididymal sperm fertilized oocytes that were collected from superovulated WT females. However, compared with WT male mice, the rate of 2-cell and blastocyst was zero in *Wdr63*-KO male mice (Fig. 6a). Intracytoplasmic sperm injection (ICSI) has been reported to be efficient for most MMAF-associated patients. To examine whether WDR63-associated male infertility could be overcome via ICSI, we conducted experiments using the sperm from WT and *Wdr63*-KO male mice. Our data show, 2-cell and blastocyst were successfully obtained upon ICSI using the spermatozoa from *Wdr63*-KO male mice. Our findings indicated that *Wdr63*-associated KO male infertility in mice could be overcome by ICSI (Fig. 6b). Consistent with above experimental observations in mice, ICSI using the sperm from Patient-M1 individual was applied and successfully resulted in a live birth. Overall, our data strongly suggest that ICSI could serve as a promising treatment for infertile men harboring bi-allelic *WDR63* variants.

## Discussion

In this study, we identified two bi-allelic variants of *WDR63* in two male infertility patients. *Wdr63*-KO male mice showed severely decreased sperm number, sperm motility and abnormal flagellar morphology. In addition, we found that WDR63 binds to WDR78 and is required for IDA assembly and flagella formation.

Notably, the two LOF variants identified in *WDR63* were either absent or rare in the human genome datasets archived in the ExAC (MAF_M1_ = 3.3 × 10^−05^, MAF_M2_ = 1.3 × 10^−04^) and gnomAD (MAF_M1_ = 1.3 × 10^−05^, MAF_M2_ = 2.9 × 10^−04^) databases without homozygous (Table 1). However, it is unclear if WDR63 variants inherited from M1 and M2 parents or spontaneous because no DNA samples of M1 and M2 consanguineous parents was available*. WDR63* encodes a predicted 891-amino-acid protein that comprises four Trp-Asp (WD40) repeat domains (398–785 aa, Uniprot ID: B2RY71). In the present study, both of the two variants in *WDR63* are located before the WD40 domain (p.Arg55* and p.Arg359*), leading to the loss of WD40 domains in WDR63 protein. The basic common function of all WD40-repeat proteins is to coordinate the assembly of multi-protein complex, where the repeating units act as a rigid scaffolds for protein interactions^22^. The complex structure of sperm flagella contained a series of WD40-domain-containing proteins^1,23^. These proteins including *CFAP43* (also known as *WDR96*, MIM: 617558), *CFAP44* (also known as *WDR52*, MIM: 617559) and *WDR66* (MIM: 618146) are associated with MMAF and their bi-allelic variants can reduce sperm motility and cause male infertility^24–26^. In our study, we found that lacking WD40 repeats domain affects the binding of WDR63 and WDR78. These findings indicated that WD40-domain-containing proteins could play an important role in cilia- and flagella-associated functions. Therefore, we speculated that the lack of the WD40 domain reduced by the LOF variants in *WDR63* may be an important factor in our MMAF patients.

In this study, we demonstrated that *Wdr63*-KO male mice is completely sterility. In addition, we noticed that a previous study reported that *Wdr63*-KO male mice with a 472 bp deletion spanning exon 2 and exon 3 exhibited normal fertility^27^. Further analysis of *Wdr63* gene transcripts, we found that *Wdr63* produces two distinct *Wdr63* mRNA isoforms differing in their transcriptional initiation, named long isoform (NM_172864.3) and short isoform (XM_017319579.3) (Supplementary Fig. S6a). Our data indicated that both of the transcripts were expressed in wild-type mice testes, suggesting that two transcripts have the potential to be translated into proteins (Supplementary Fig. S6b, c). Thus, we engineered a sgRNA target on exon 10 of *Wdr63*, which located at common regions both of the transcripts (Supplementary Fig. S6a). Besides, the off-target evaluation of CRISPR-Cas9 suggested that the two genotypes *Wdr63*-KO mice were successfully on-target (Supplementary Fig. S7). Therefore, we speculate that the phenotype of *Wdr63*-KO male sterility can only be caused when two transcripts are absent at the same time.

WDR63 and WDR78 are subunits of IDA intermediate chains in mammalian. The IDAs are highly conserved from unicellular protozoa and sperm to the mammalian ciliated epithelia of the respiratory and reproductive tracts^21^. Most studies have shown IDAs are both necessary and sufficient to cilia and flagella bend formation and beating form in *Chlamydomonas*^16^. Notably, variants or knockdown of IDA complex proteins result in severe asthenoteratozoospermia and/or primary ciliary dyskinesia (PCD) in mammalian. For instance, the first report MMAF-associated gene *DNAH1* is component of monomeric IDA heavy chains, and variants in *DNAH1* result in damage of IDA without PCD^18^. Furthermore, variants in IDA-associated proteins *DNAH2* (MIM: 603333), *DNAH6* (MIM: 603336) can cause severe sperm flagella defects that damage sperm motility^28,29^. In our study, WDR63 is the vertebrate orthologue of IC140, a subunit of IDA intermediate chains in *Chlamydomonas*, indicating its IDA location and potential function in human and mice sperm flagellar. Previous studies revealed that IC140 is essential for the flagellar assembly of *Chlamydomonas*, but it is poorly known in mammalian^30,31^. We found that the expression of DNAH10, DNAH2 and WDR78 were significantly decreased in *Wdr63*-KO mice, demonstrating the important roles of WDR63 in flagellar assembly in mammalian. Notably, we performed immunoprecipitation with testicular extracts using anti-WDR63 antibodies, mass spectrometric analysis only identified WDR78 is the potential associated proteins. Further co-IP experiments confirmed that WDR78 is associated with WDR63. Immunofluorescence analysis also found the co-localization of WDR63 and WDR78. These experimental observations indicate that WDR63 interacts with WDR78 and is necessary for IDA assembly.

Presently, assisted reproductive technology (ART), such as IVF and ICSI, expanded opportunities for infertile couples^32^. Previous studies have suggested that MMAF-affected men harboring *DNAH1*, *DNAH8* or *TTC29* (MIM: 618735) variants could have success clinical outcomes following ICSI^18,33,34^. In contrast, *CEP135* (MIM: 611423) and *DNAH17*-associated MMAF men have a failed pregnancy^2,35^. In this study, although IVF experiments failed to obtain the two-cell embryo and blastocyst from *Wdr63*-KO male mice, the rate of two-cell embryo and blastocyst with ICSI experiments were normal in *Wdr63*-KO male mice compared with WT male mice. This experiment suggesting that male infertility of *Wdr63*-KO mice could be overcome by ICSI. Furthermore, MMAF-affected men who harboring *WDR63* variants underwent ICSI treatment acquired successful clinical pregnancy and obtained a biological offspring. Therefore, our findings demonstrate that ICSI can be recommended for WDR63-associated MMAF.

In conclusion, we identified that bi-allelic variants of *WDR63* cause male infertility via abnormal inner dynein arms assembly and flagella formation. Our finding will be informative for clinical decision making and appropriate genetic counseling of MMAF.

## Materials and methods

### Human samples

The first cohort of 243 MMAF-affected Chinese men recruited from the Reproductive and Genetic Hospital of CITIC-Xiangya (Changsha, China). Subjects with evident PCD-related symptoms, such as recurrent airway inflammation, bronchiectasis, and otitis media, were excluded. Patients presenting with other causes of infertility, such as reproductive malformation, drug use, and exposure to gonadotoxic factors, were also excluded. The second cohort of 121 NOA-affected Chinese men was enrolled from the First Affiliated Hospital of Nanjing Medical University (Nanjing, China). Those with a history of cryptorchidism, vascular trauma, orchitis, obstruct of the vas deferens, abnormalities in chromosome number or microdeletions of the azoospermia factor region on the Y chromosome were excluded from the study. This study was approved by the institutional review boards at all the participating institutes. Signed informed consents were obtained from all subjects participating in the study.

### Semen analysis and sperm morphological analysis

Semen analysis was performed in the source laboratories during routine biological examination of the individuals according to the World Health Organization (WHO) guidelines^19^. Semen samples from the men harboring *WDR63* variants were collected through masturbation after 2–7 days of sexual abstinence and evaluated after liquefaction for 30 min at 37 °C. Analyses of semen volume, sperm concentration, and motility were carried out and replicated in the source hospitals during routine examination.

Semen samples from mice were collected from the cauda epididymides obtained through dissection of adult male mice and incubated in human tubal fluid (HTF) medium (Millipore, Cat. # MR-070-D) supplemented with 10% FBS at 37 °C for 5 min. Sperm count and motility assessment were performed using the Computer Assisted Sperm Analyzer (CASA) system.

### Whole-exome sequencing (WES) and Sanger sequencing

We performed a two-stage sequencing analysis. Genomic DNA was isolated from peripheral-blood samples of the subjects using a whole blood DNA purification kit (Qiagen, Hilden, Germany). At the first stage, Whole-exome sequencing (WES) was performed on MMAF-affected subjects as previously described^4^. Briefly, genomic DNA sequencing libraries were prepared with the Nextera Rapid Capture Exome sample preparation kit, targeting >214,000 coding exons and following the protocol provided by the manufacturer (Illumina). Nextera Rapid Capture Exome libraries were sequenced on HiSeq2000 with 100-bp paired-end reads. FastQ files were subsequently trimmed and aligned to the human reference genome (hg19, GRCh37) with the Burrows-Wheeler Aligner (BWA, v.0.6.1)^36^. For each sample, the mapping efficiency of generated WES reads was 99% and the coverage of exonic regions with ≥10 reads was 93%. Single-nucleotide substitutions and small indel variants were called with Genome Analysis Toolkit 1.6-7-g2be5704 (GATK)^37^. Variant sites >10 bp away from the nearest exome sequence capture target or with low quality score (<Q20) were filtered out. ANNOVAR was performed for functional annotation through a variety of databases, such as ExAC and gnomAD^32^. After filtering, the retained variants were submitted to PolyPhen-2, SIFT, Mutation Taster and CADD for functional prediction^38–40^. The second stage, candidate pathogenic gene from WES sequencing were validated by Sanger sequencing with another 121 NOA-affected subjects. PCR amplification was performed with Dyad Polymerase (Bio-Rad Laboratories). DNA sequencing of PCR products was conducted on an ABI377A DNA sequencer (Applied Biosystems). The primers for PCR are listed in Supplementary Table S2.

### Screening process of harboring variants in *WDR63* gene

To identify the potential MMAF-associated variants in male infertility, we first defined testis-high expression genes (THG) according to our previous study^41^. Briefly, a total of 20,719 unique genes with expression abundance data (FPKM) from three datasets (GTEx, Illumina Human Bodymap and NJMU-seq) were included in the analysis after integration. As a result, we identified 1606 THGs characterized with Specificity measure values >0.8 (SPM_GTEx_ > 0.8, SPM_HBM_ > 0.8, SPM_NJMU_ > 0.8) and highly mRNA expression (FPKM > 5). Base on above results, we identify 539,641 variants from 243 MMAF-affected Chinese men with whole-exome sequencing (WES) datasets. According to previous pedigree analyses, MMAF has been assumed to follow an autosomal recessive inheritance^25,34^. Therefore, we mainly focused on bi-allelic rare variants depend on variants frequency and functional annotation, and variants minor allele frequency (MAF) ≥ 0.001 in the gnomAD database and combined annotation-dependent depletion (CADD) score of < 15 were filtered out. Nonsense, frameshift, and essential splice-site variants were preferred. Missense variants predicted to be deleterious simultaneously by the bioinformatics tools of SIFT, PolyPhen-2, and/or Mutation Taster were also included for further evaluation. Finally, 18 candidate genes with 43 bi-allelic loss-of-function variants were retained and validated by Sanger sequencing. Among these genes, *CFAP65*, *CFAP47*, *FSIP2*, *CFAP43* and *TTC21A* have been reported in other study^4,11,22,25,42^. Inner dynein arm (IDA), composed of a series of protein complex, is necessary to cilia and flagella bend formation and beating^16^. Previous studies indicated defects of IDA protein complex result in multiple morphological abnormalities of the sperm flagellum (MMAF) and male infertility^29,43,44^. Thus, we selected the IDA-associated protein, WDR63, from remaining 13 candidate genes for the follow-up study (Supplementary Fig. S1).

### Generation of *Wdr63* knockout mouse models

*Wdr63* knockout mouse model was created by the animal center of Nanjing Medical University (Nanjing, China). Briefly, single guide RNA (sgRNA: CCGTGCGCCTCTCCTATGAAGAG) was designed to target exon 10 of the *Wdr63*. The oligonucleotide used to generate the sgRNA expression plasmid was annealed and cloned into the BsaI site of pGL3-U6-sgRNA-PGK-puromycin (Addgene 51133). Transcription and microinjection of CRISPR/Cas9 was performed in vitro^45^. Briefly, the Cas9 plasmid (pST1374-Cas9-N-NLS-Flag-linker, Addgene 44758) was linearized with AgeI and transcribed using the T7 Ultra Kit (Invitrogen), followed by purification using RNeasy Mini Kit (Qiagen). pUC57-sgRNA expression vectors were linearized by DraI and transcribed using the MEGAshortscript Kit in vitro (Invitrogen). sgRNAs were purified by MEGAclear Kit (Invitrogen). Mixture of Cas9 mRNA (20 ng/μl) and two sgRNAs (5 ng/μl each) were injected into the cytoplasm and male pronucleus of the zygote by electroporation. Embryos were implanted into pseudo-pregnant C57BL/6J females according to standard procedures. Founder mice were backcrossed to C57BL/6 J. The off-target evaluation of CRISPR Cas9 suggested that the *Wdr63*-KO mice were successfully on target (Supplementary Fig. S3). All animal studies were approved by the Institutional Animal Care and Use Committee of Nanjing Medical University, Nanjing, China. The primer used for *Wdr63*-KO mouse genotype analysis are listed in Supplementary Table S3. Primers used for off-target evaluation are listed in Supplementary Table S4.

### WDR63 combinant protein and antibody generation

The full-length coding sequence of the *Wdr63* was subcloned into a pReceiver-B01 (OmicsLinkTM) expression vector (GeneCopoeia, MD, USA) coding for six N-terminally located histamine residues and expressed in BL21 (DE3) pLysS competent cells according to the manufacturer’s instructions. The expressed recombinant protein was then purified over a Ni^2+^ affinity column using the AKTA Basic System (Amersham Biosciences) under denaturing conditions. Polyclonal antibodies were produced in a male New Zealand white rabbit by injecting 100 mg of recombinant protein and boosting with one-half of the initial amount at 2 and 3 weeks after the primary immunization. Reactivity of the pre-immune and immune sera was determined by ELISA.

### Real-time quantitative PCR (qPCR) analyses

Total RNA of mice tissues was extracted using TRIzol reagent (Invitrogen) and was converted to cDNA using a RevertAid First-Strand cDNA Synthesis Kit (ThermoFisher). Real-time PCR was performed using SYBR Premix Ex Taq II (TaKaRa) on an iCycler RT-PCR Detection System (Bio-Rad Laboratories). The ΔΔCT method was used for data analysis. Each assay was performed in triplicate for each sample. The *Gapdh* gene was used as an internal control. The primers for real-time PCR are listed in Supplementary Table S5.

### Immunofluorescence (IF)

For immunofluorescence assay, moue spermatozoa samples were fixed onto slides with 4% paraformaldehyde, permeabilized with 0.5% TritonX-100 in PBS, and blocked with 5% BSA. Next, The slides were sequentially incubated overnight at 4 °C with the following primary antibodies: anti-WDR63 (described above, 1:1000), anti-WDR78 (sc-390633, Santa Cruz, 1: 50), anti-Acetyl-α-Tubulin (D20G3, CST, 1:1000). The slides were washed in 1× PBS, incubated with Alexa Fluor 488 (1:500, A21206, Thermo Fisher) or Alexa Fluor 594 (1:500, A11005, Thermo Fisher) labeled secondary antibodies for 2 h at room temperature, and then counterstained with Hoechst 33258 to label the nuclei. Images were acquired using a laser scanning confocal microscope (LS800).

### Coimmunoprecipitation (Co-IP)

The extracted proteins were incubated with 3 μg of target antibodies overnight at 4 °C. Next, 50 μl of Protein A/G magnetic beads (LSKMAGAG10, Millipore) was added to each incubation sample for 1 h at room temperature. The beads were washed three times with 1× PBS. Finally, the coimmunoprecipitated proteins were eluted by standard 1× SDS sample buffer and heated for 10 min at 70 °C and then separated on 10% SDS-polyacrylamide gels and PVDF membranes for the immunoblot analysis.

### Histological analysis

For H&E (hematoxylin and eosin) staining, the epididymis was fixed in modified Davidson liquid (30% of a 37–40% formaldehyde stock solution, 15% ethanol, 5% glacial acetic acid, and 50% distilled water) and embedded in paraffin. Sections were cut at a 5 μm thickness. The sections were then dewaxed with xylene, hydrated, H&E stained, dehydrated with ethanol (70%, 80%, 90%, 100%) and finally blocked with resin. For periodic acid–Schiff (PAS) staining, testes are also fixed in MDF and embedded in paraffin. The sections were then deparaffinized, hydrated, stained with Periodic Acid–Schiff reagent, counterstained with hematoxylin, dehydrated and blocked.

### Immunoblot analysis

The proteins of the cultured cells and mice testes tissues were extracted using a universal protein extraction lysis buffer (Bioteke) containing a protease inhibitor cocktail (Roche). The denatured proteins were separated on 10% SDS-polyacrylamide gels and transferred to a polyvinylidene difluoride (PVDF) membrane (Millipore) for the immunoblot analysis. The antibodies used in western blotting, Co-IP and immunofluorescence staining are as follows: anti-WDR63 (described above, 1:1000), anti-WDR78 (sc-390401, Santa Cruz, 1:100), anti-DNAH2 (HPA067103, Sigma-Aldrich, 1:1000), anti-DNAH10 (bs-11022R, Bioss, 1:1000), anti-HA-tag (M180-3, MBL, 1:1000), anti-Flag-tag (F3165, Sigma-Aldrich, 1:1000).

### Scanning and transmission electron microscopy

For scanning electron microscopy (SEM), the cauda epididymides obtained through dissection of adult male mice and incubated in human tubal fluid (HTF) medium (Millipore) supplemented with 10% FBS at 37 °C for 5 min. The sperms were fixed at 4 °C overnight with 2.5% glutaraldehyde in 0.2 M cacodylate buffer (50 mM cacodylate, 50 mM KCl, and 2.5 mM MgCl2, pH 7.2). After immersed in 1% OsO_4_ in 0.2 M cacodylate buffer for 2 h at 4 °C, the specimens were dehydrated through a graded ethanol series dried through the use of a CO_2_ critical-point dryer (Eiko HCP-2, Hitachi). Afterward, the specimens were mounted on aluminum stubs, sputter coated through using an ionic sprayer meter (Eiko E-1020, Hitachi), and analyzed via SEM (Stereoscan 260).

For transmission electron microscopy (TEM), the adult mouse cauda epididymis was fixed at 4 °C overnight with 2.5% glutaraldehyde in 0.2 M cacodylate buffer. After washing in cacodylate buffer, the specimens were cut into small pieces of approximately 1 mm^3^ and immersed in 1% OsO4 in 0.2 M cacodylate buffer for 2 h at 4 °C. Next, the samples were washed and submerged in 0.5% uranyl acetate overnight, dehydrated through a graded ethanol series, and embedded in resin (Low Viscosity Embedding Media Spurr’s Kit, EMS, 14300). Ultrathin sections were cut on an ultramicrotome and mounted on copper grids. The sections then were stained with uranyl acetate and lead citrate for 10 min and observed using a JEM-1400 transmission electron microscope (JEOL).

### In vitro fertilization (IVF) and intracytoplasmic sperm injection (ICSI)

Two-month-old B6D2F1 (C57BL/6 3 DBA2) wild-type female mice were superovulated by injecting 5–7.5 IU of pregnant mare serum gonadotropin (PMSG), followed by 5–7.5 IU of human chorionic gonadotropin (hCG) 48 h later. For IVF, wild-type and homozygous sperm samples were collected from mouse cauda epididymis incubated in human tubal fluid (HTF) medium drop. Then cumulus-intact oocytes, collected from superovulated females, were transferred into the sperm-containing HTF drop. After 5–6 h of incubation, the embryos were washed in HTF and transferred into KSOM medium (Millipore, Cat. # MR-106-D) to further culture at 37 °C under 5% CO2. Fertilization rates were evaluated by recording the numbers of two-cell embryos and late-stage blastocysts at 20 h and 91 h later, respectively. For ICSI, oocytes were obtained from superovulated females, and sperm heads were injected into oocytes through using a Piezodriven pipette. Then the injected oocytes were cultured inKSOM medium at 37 °C under 5% CO2. Two-cell embryos and blastocysts were counted 20 h and 96 h later, respectively.

### Statistical analysis

Numerical data are presented as means ± SEM. The statistical significance of the difference between the mean values for the different genotypes was examined using Student’s *t*-test with a paired two-tailed distribution. The data were considered significant when *P* ≤ 0.05 (*) and 0.01(**).

## Supplementary information

Supplementary information

Supplementary Movie S1

Supplementary Movie S2

Supplementary Data S1

## Data Availability

All relevant data are available from the corresponding author. Source data are provided with this paper.
