# Supplementary material for: Bi-allelic variants in human WDR63 cause male infertility via abnormal inner dynein arms assembly
Source: Cell Discov. 2021 Nov 16;7:110. doi: 10.1038/s41421-021-00327-5 (PMC8593051; doi:10.1038/s41421-021-00327-5)
Supplement: Supplementary file 1 — Supplementary information [file 41421_2021_327_MOESM1_ESM.docx]

**Supplementary Information
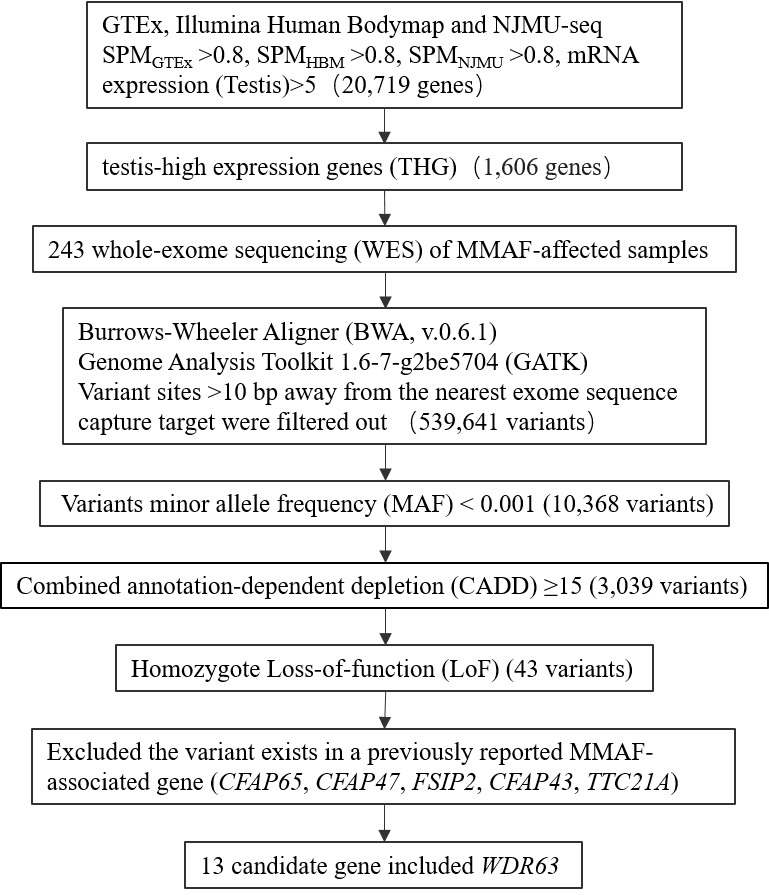
**

**Supplementary Fig. S1: The screening process of harboring variants in *WDR63* gene.**

**
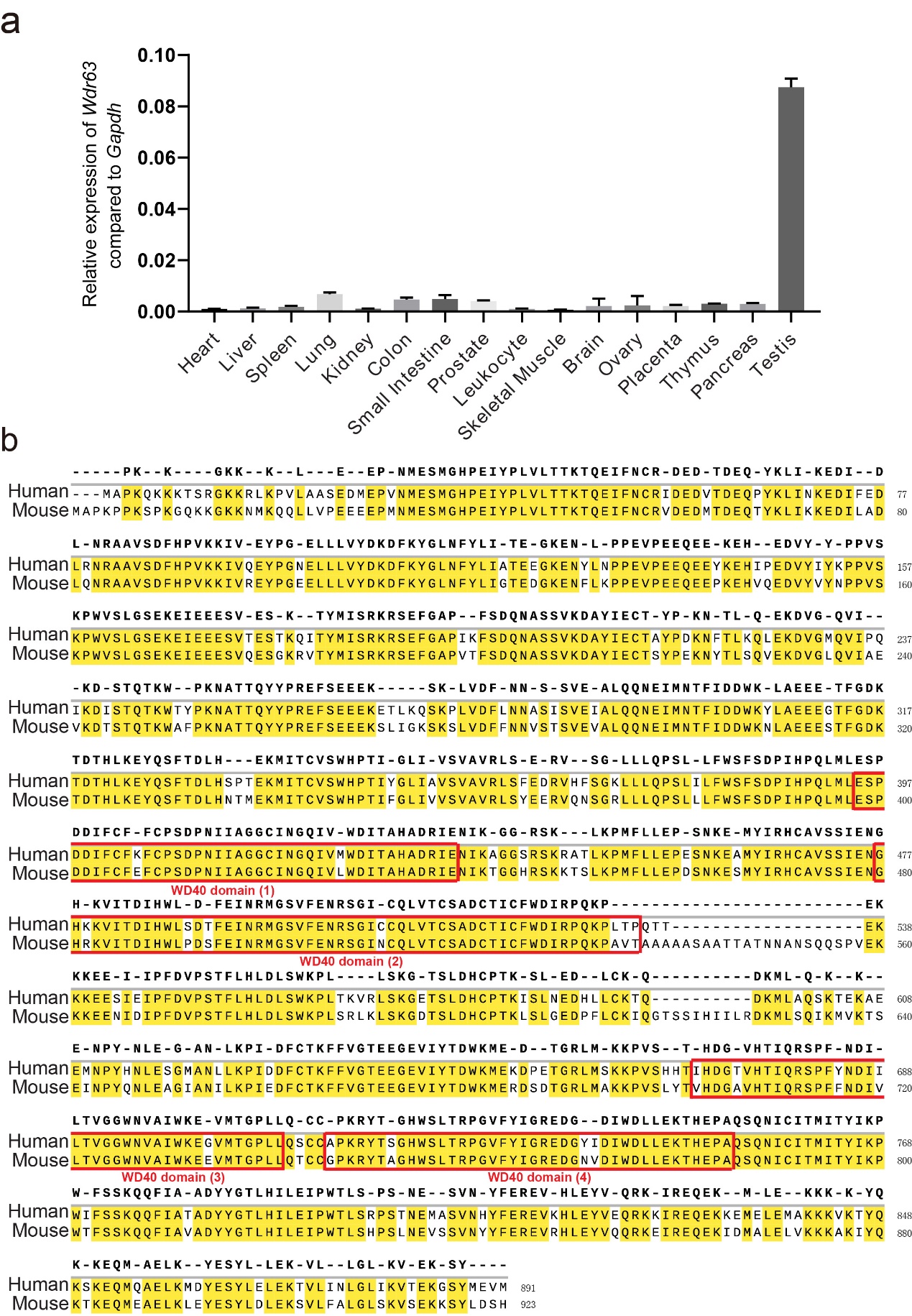
**

**Supplementary Fig. S2: WDR63 was highly expressed in human testes and conserved among different species**

**a** Real-time q-PCR for *Wdr63* in various human tissues with *Gapdh* as a control. Human tissues cDNA come from Human MTC Panel I kit (Cat. #: 636742, Clontech). Error bars, SEM (n = 3). **b** Amino acid sequence alignment between human and mouse WDR63 protein. The red box indicates WD40 domain of WDR63 in human and mouse.

*
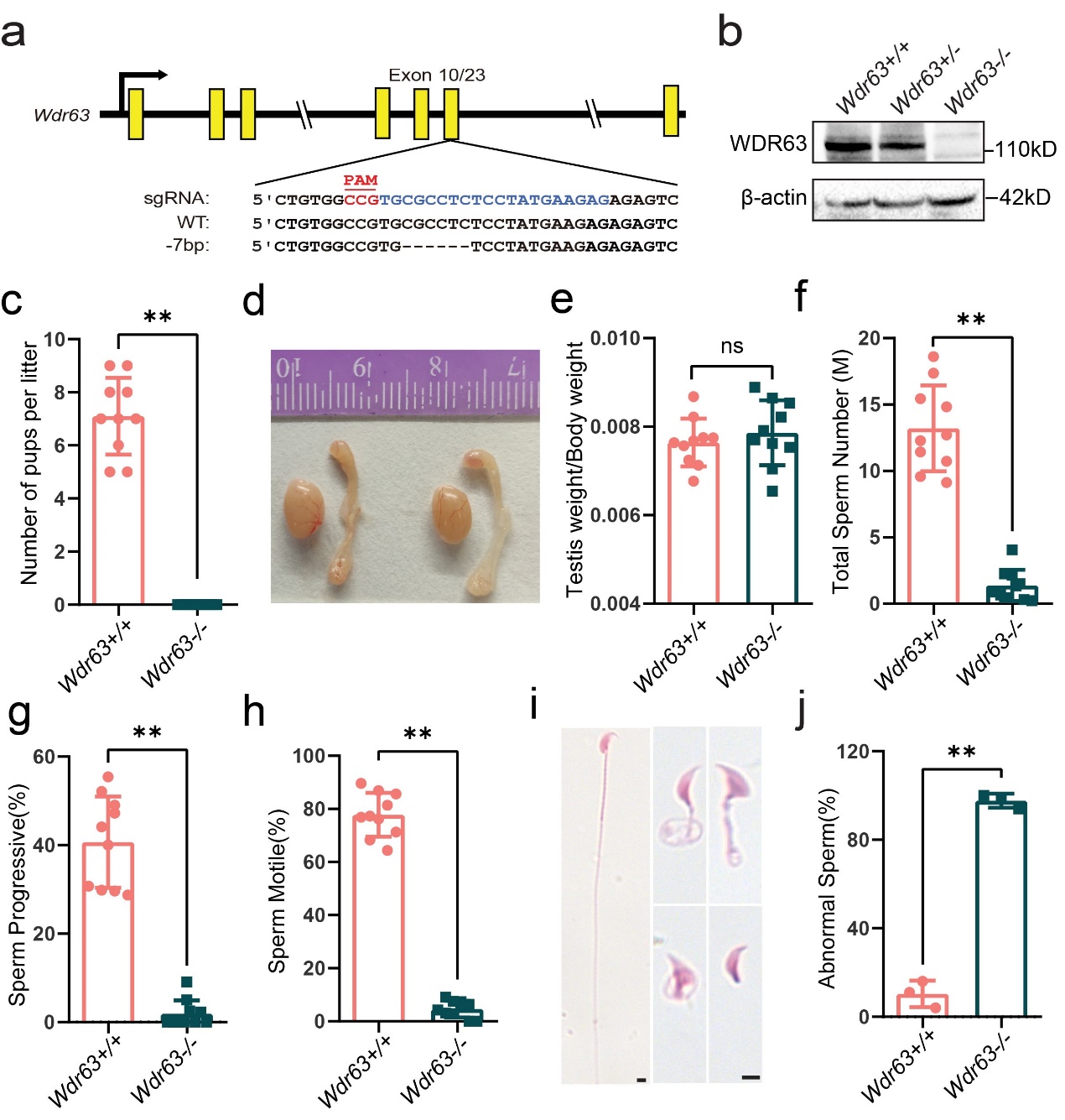
*

**Supplementary Fig. S3: Phenotypes of *Wdr63* null male mice with 7-bp frameshift deletion**

**a** Schematic of the *Wdr63* knocked out mouse utilized CRISPR/Cas9 system. The sgRNA was designed to target exon 10 of the *Wdr63*, a 7-bp frameshift deletion was obtained. **b** Western blot analysis of *Wdr63* wild-type (*Wdr63−/−*), heterozygous mice (*Wdr63+/−*) and homozygous mice (*Wdr63*+/+) testes with a mouse polyclonal antibody raised against WDR63. β-actin was used as a loading control. **c** Breeding results from *Wdr63*+/+and *Wdr63*−/−. **d-e** Testes and [epididymis](javascript:;) from 8-week-old mice were comparable between *Wdr63*+/+ and *Wdr63−/−* male mice. **f** Total sperm number, **g** sperm progressive and **h** sperm motile were analysis by Computer Assisted Sperm Analyzer (CASA) method. **i-j** HE staining analysis morphology of the spermatozoon from *Wdr63+/+* and *Wdr63*−/− male mice evident under light microscopy. Scale bars: 5 μm. All the phenotypes consistent with *Wdr63* null Male Mice with the nonsense mutation (GCG-TGA) plus a 2-bp deletion genotype. Abbreviations: M, million. For **c** to **h**, n=10 and the bars represent mean ± SEM. For **j**, n=3 and the bars represent mean ± SEM. The statistical analysis was carried out using One-way ANOVA test, ** denotes *P* < 0.01; ns, not significant.


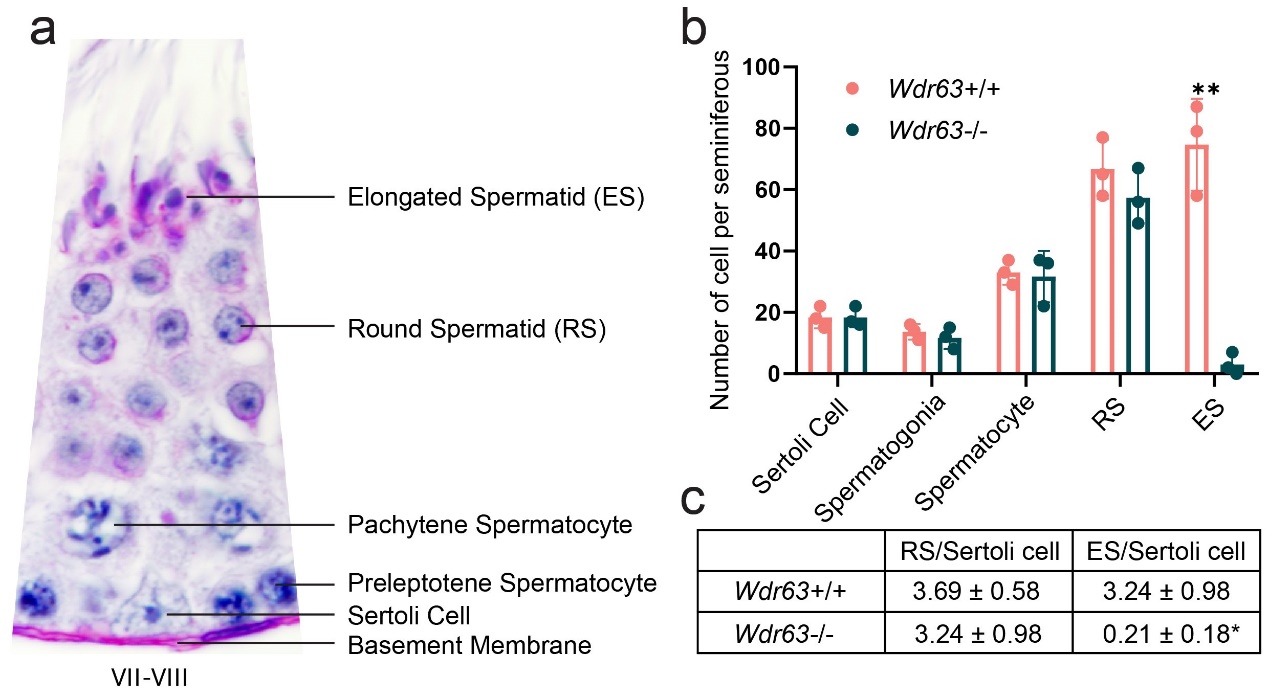


**Supplementary Fig. S4: The number of elongated spermatids is significantly reduced in VII–VIII seminiferous tubules.**

**a** Schematic diagram of different spermatogenic and sertoli cells in Periodic acid–Schiff (PAS) staining. **b** The number of different spermatogenic and sertoli cells per seminiferous. **c** the ratios of elongated spermatid and Sertoli cells in homozygous and wild-type. For **b** to **c**, n=3 and the bars represent mean ± SEM. The statistical analysis was carried out using One-way ANOVA test, * denotes *P* < 0.05, ** denotes *P* < 0.01.


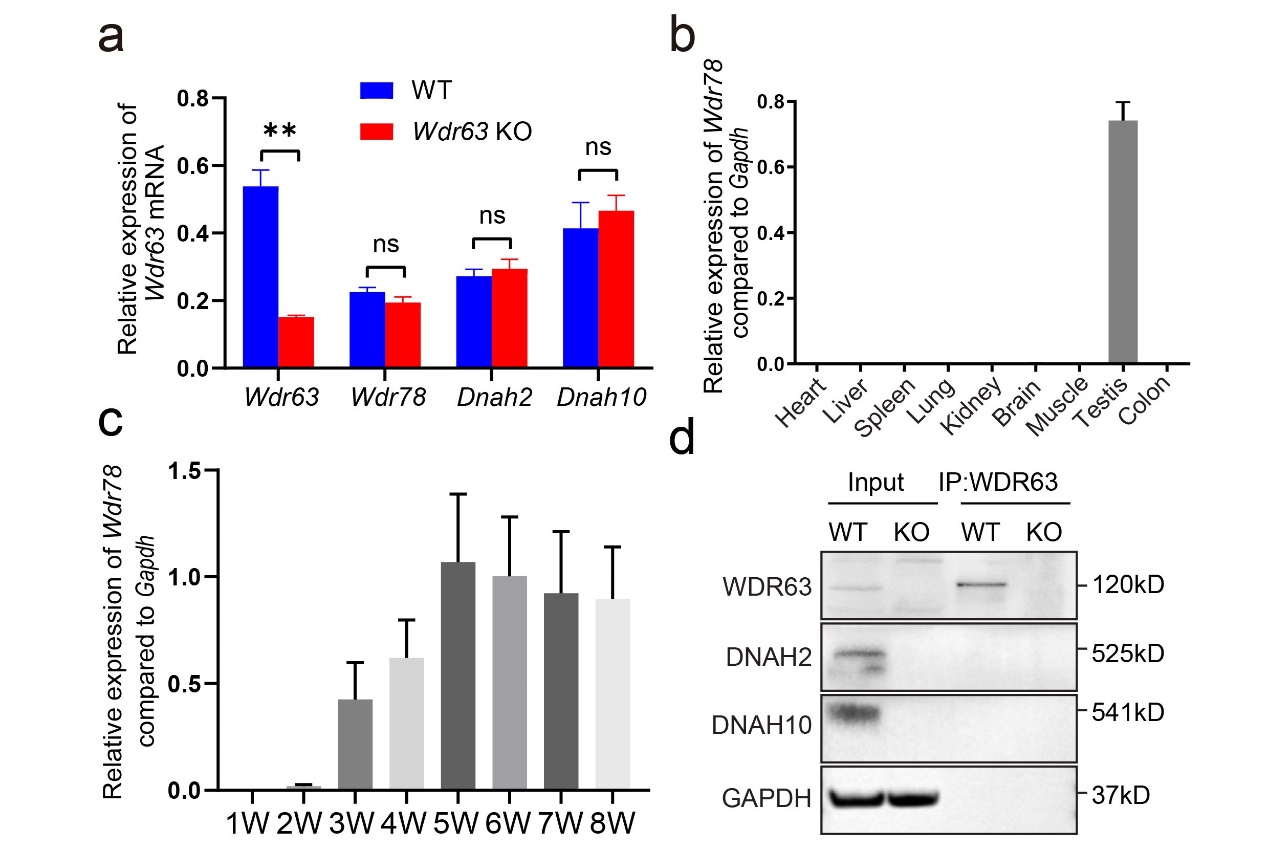


**Supplementary Fig. S5: WDR63 only bind to WDR78 but not DNAH2 and DNAH10 in mammals**

**a** mRNA levels of *Wdr63*, *Wdr78*, *Dnah2* and *Dnah10* in wild-type and *Wdr63*-KO mouse testis. **b** Real-time q-PCR for *Wdr78* transcripts in various mouse tissues, and *Gapdh* gene is used as a control. Error bars, SEM (n = 3). **c** mRNA levels of *Wdr78* in mouse testis at the indicated time points. Error bars, SEM (n = 3). **d** Immunoblotting followed by co-IP assays demonstrated that WDR63 did not bind to DNAH2 and DNAH10 in mammals. For **a**, n=3 and the bars represent mean ± SEM. The statistical analysis was carried out using One-way ANOVA test, ** denotes *P* < 0.01; ns, not significant.


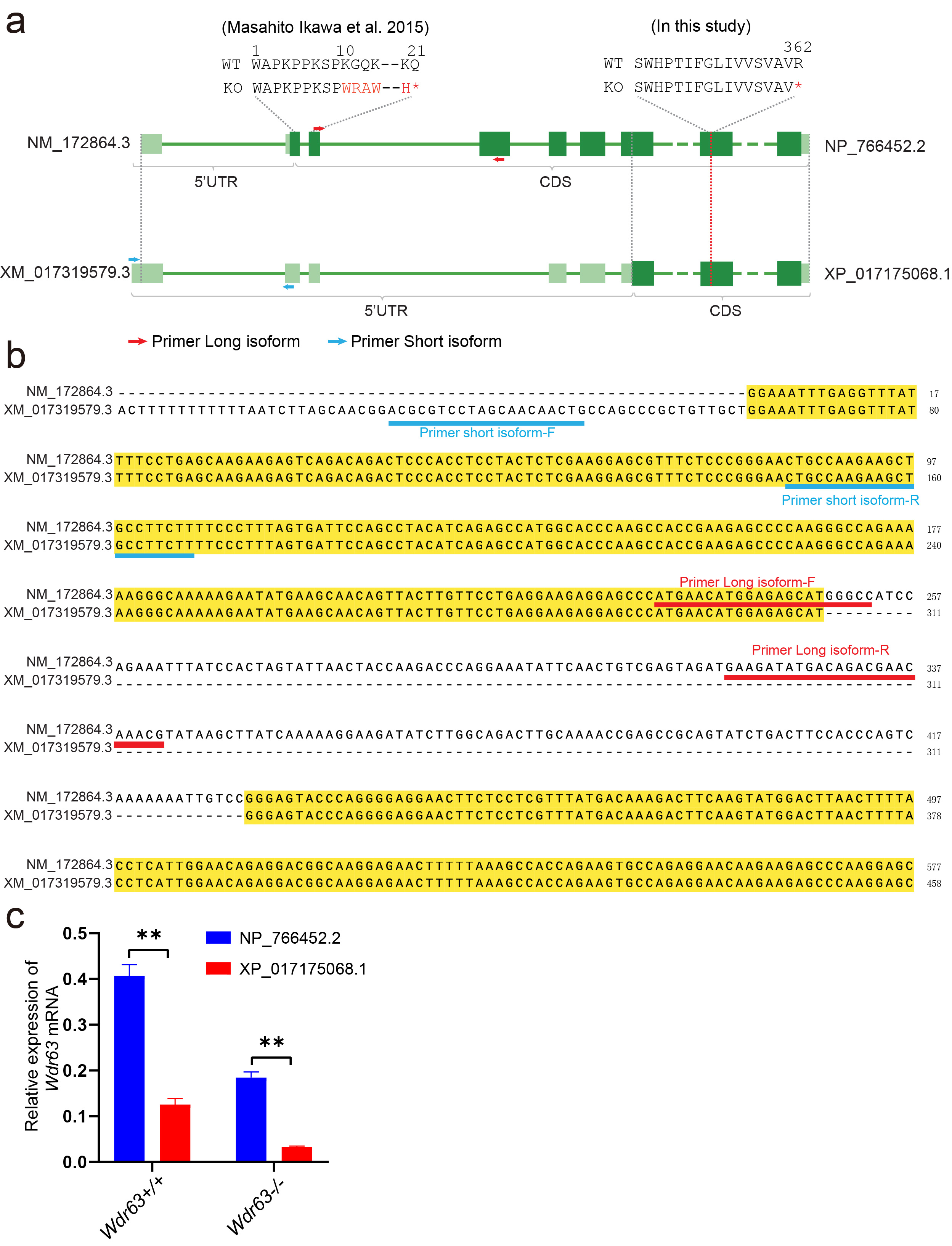


**Supplementary Fig. S6: *Wdr63* produce two distinct *Wdr63* transcripts and both of them expressed in mice testes.**

**a** As show in schematic diagram, *Wdr63* produce two distinct *Wdr63* mRNA isoforms differing in their transcriptional initiation, named long isoform ([NM_172864.3](https://www.ncbi.nlm.nih.gov/nuccore/NM_172864.3)) and short isoform ([XM_017319579.3](https://www.ncbi.nlm.nih.gov/nuccore/XM_017319579.3)). A previous study reported that *Wdr63*-KO male mice with a 472bp deletion spanning exon 2 and exon 3 induced a premature stop codon in long isoform but not short isoform. In this study, we engineered a sgRNA target on exon 10 of *Wdr63*, which located at common regions of two transcripts. Two pairs of primers in non-common regions were used to distinguish long and short transcripts. **b** Sequencing alignment of non-common regions between long and short transcripts. The underlining indicates the location of primers at two transcripts. **c** Real-time q-PCR for two transcripts of *Wdr63* in wild-type and *Wdr63*-KO mice testes, and *Gapdh* gene is used as a control. For **c**, n=3 and the bars represent mean ± SEM. The statistical analysis was carried out using One-way ANOVA test, ** denotes *P* < 0.01.


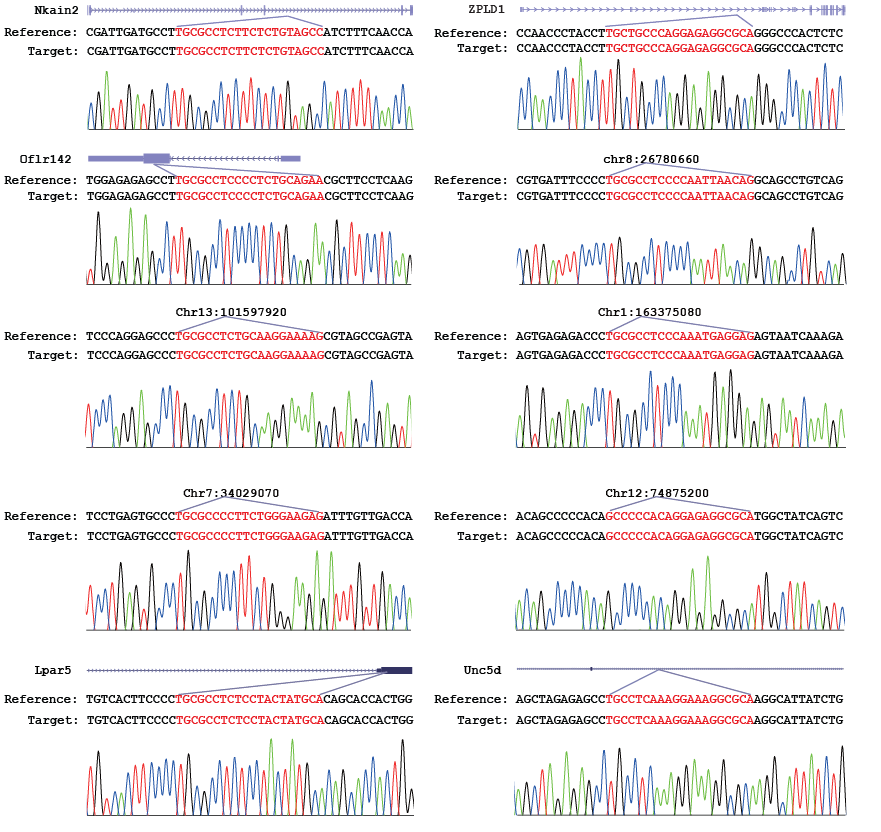


**Supplementary Fig. S7: The off-target evaluation of CRISPR-Cas9 in *Wdr63*-KO mice**

The potential off-targets site's genomic locations were identified by Off-Spotter server. Sanger sequencing using to verify the sequence of off-targets sites is mismatch.

**
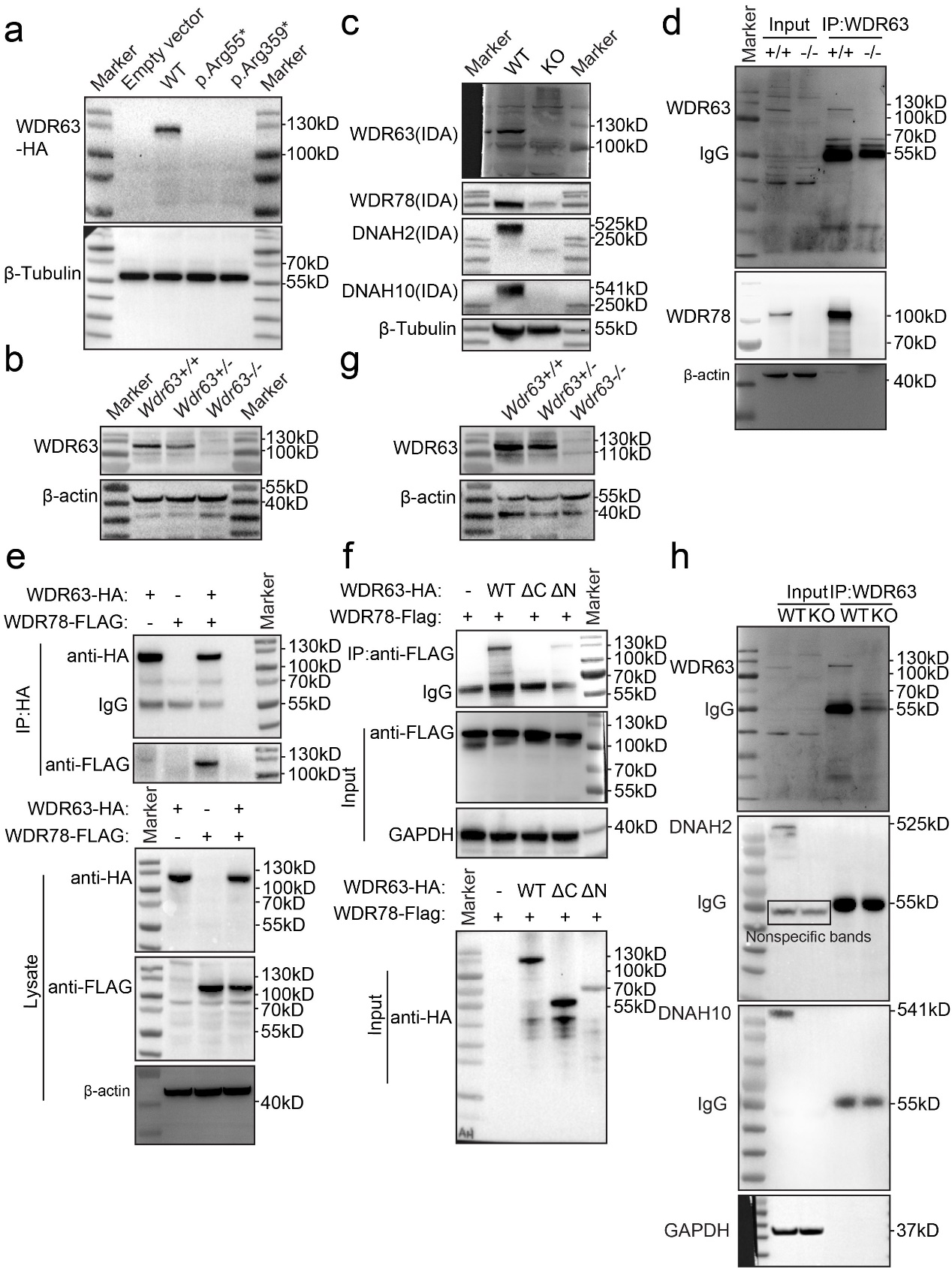
**

**Supplementary Fig. S8: Original Immunoblots for Indicated Figures**

**a-h** relative to Fig. 1d, Fig. 2d, Fig. 5a, Fig. 5b, Fig. 5c, Fig.5f, Supplementary Fig. 3b, Supplementary Fig. 5d, respectively.

**Supplementary Tables**

| **Supplementary Table S1: Semen characteristics of men carrying bi-allelic *WDR63* variants** | | | |
| --- | --- | --- | --- |
|  | M1 | M2 | Reference Values |
| **Semen Parameters** | | | |
| Semen volume (mL) | 1.6 | 1.5 | >1.5 |
| Sperm concentration (10^6^/mL) | 7.5^*^ | 0^*^ | >15.0 |
| Total sperm count (10^6^) | 12.0^*^ | 0^*^ | >39.0 |
| Motility (%) | 0^*^ | 0^*^ | >40.0 |
| Progressive motility (%) | 0^*^ | 0^*^ | >32.0 |
| Lower and upper reference limits are shown according to the World Health Organization standards and the distribution ranges of morphologically abnormal spermatozoa observed in fertile individuals.  *Abnormal values. | | | |

| **Supplementary Table S2: Primers used for WDR63 Sanger sequencing.** | |
| --- | --- |
| **Primer Name** | **Primer Sequence (5'-3')** |
| WDR63-exon1-F | AGAGTTGCTGCGGTTTGTG |
| WDR63-exon1-R | GCCTTTCCCATTCAGAGCTT |
| WDR63-exon2-F | GCCCAGGGTAAATCTTAGAAAAC |
| WDR63-exon2-R | AAAAATCCCATTCAATGAAGAAATA |
| WDR63-exon3-F | TGATTCAAAAATGTAAATTTGACTTCC |
| WDR63-exon3-R | TCAGTTTGTAGTATTCCTGCTTGC |
| WDR63-exon4/5-F | AGGACCTGCGCAACAGAG |
| WDR63-exon4/5-R | ACAAGCAGAAGCTCATTTCCA |
| WDR63-exon6-F | GCTATAACCTAAACTTGGGCATACA |
| WDR63-exon6-R | CCCTCAGGAATAAGTGTTGTTCA |
| WDR63-exon7-F | TCAGGGACTTCATTATGTTACCTTT |
| WDR63-exon7-R | CATTTTGTCTGAGTGCTTATGTCC |
| WDR63-exon8-F | TCATGTAGCCTTTGAATAGTGGTC |
| WDR63-exon8-R | TGCATTGTTAAGAAAATCAACCA |
| WDR63-exon9/10-F | CTGTGTCTCATGGCATCCAA |
| WDR63-exon9/10-R | AAAAGAAAGTCGCACGGCTA |
| WDR63-exon11-F | TCGCCATGATCTTAATCTCTTTT |
| WDR63-exon11-R | GTCCAAGGAACTGCCAAGTC |
| WDR63-exon12/13-F | AAGAGCCACACTGAAGGTAAGC |
| WDR63-exon12/13-R | CATTTTCTATTGAAGAGACTGCACA |
| WDR63-exon14-F | CGGCCGGTATGTTCTTTTATT |
| WDR63-exon14-R | GCAACAATCCATAACATTCTTTCA |
| WDR63-exon15-F | AGGCTGCACCTCTTGTTTGT |
| WDR63-exon15-R | TGAGCTGCTCTACTTTTAGAGAGTGT |
| WDR63-exon16-F | TCTTTGCTATATGTTCTCCCAAAA |
| WDR63-exon16-R | ACCGTGTTAGCCAGGATGAT |
| WDR63-exon17-F | GCAAGGGGCAGAAAATGTAA |
| WDR63-exon17-R | TGCAAACAAGCATACTAAAATATGAAA |
| WDR63-exon18-F | GCACATGAGTGTGAAGTCTTTTG |
| WDR63-exon18-R | CCTCCCATTAGCAAGACCAG |
| WDR63-exon19-F | CACTGCAGGAATGCAAACAG |
| WDR63-exon19-R | AAAAAGTGTCCCAAAGGCACTA |
| WDR63-exon20-F | GCATGGTGAATTTCCCTCAG |
| WDR63-exon20-R | TGTTCCCGAGTTACCCAAAA |
| WDR63-exon21-F | GATGGTTTTCTGTTTGCTGATTT |
| WDR63-exon21-R | TCCCTTACAGAGCAAAAATGC |
| WDR63-exon22-F | CCTGTCACCCTCTCATGGAC |
| WDR63-exon22-R | CCACTGGTGTTCGAGGAAA |
| WDR63-exon23-F | CCCCCAGAAAACATATCAGAAG |
| WDR63-exon23-R | AGAGGGAAGAAGTCCCCAAA |

| **Supplementary Table S3: Primers used for *Wdr63* KO mouse genotype analysis.** | |
| --- | --- |
| **Primer Name** | **Sequence (5'-3')** |
| WDR63-F | AACCTTCCAAGTACACTGT |
| WDR63-R | GGTAATTGATGGAGGTTGC |

| **Supplementary Table S4: Primers used for off-target evaluation.** | |
| --- | --- |
| **Primer Name** | **Primer Sequence (5'-3')** |
| chr2:90252087-90252509-F | GGGGTTCAGCATAGGGGTA |
| chr2:90252087-90252509-R | TAAAGGTGTCAGTGCAGGCA |
| chr8:26780453-26780875-F | CCTTATGGGTCCGCACAG |
| chr8:26780453-26780875-R | CTCTCCGTCCCACTCTGC |
| chr13:101597715-101598137-F | GGGCTGTGGACACTCAAAGT |
| chr13:101597715-101598137-R | CTTCCCAGAGCCTAATGTTGG |
| chr1:163374875-163375297-F | AACCCAGTGAGAGGCAGATAGA |
| chr1:163374875-163375297-R | ACACACATGCACACATGTAAACA |
| chr7:34028865-34029287-F | CTATGGTGATGGGAGCCACT |
| chr7:34028865-34029287-R | ACTAGCCCCAAAATGGCTGT |
| chr12:74874991-74875413-F | CTCGCTGGGTGGTAATTTTC |
| chr12:74874991-74875413-R | AAACCCATCCAGGGAATTAAA |
| chr6:125081392-125081814-F | CTGGTATTGGCGACTGGTCT |
| chr6:125081392-125081814-R | CCAGATGAACATGTACGGCA |
| chr8:28850810-28851232-F | AGCCACAGAACCCATAGGAA |
| chr8:28850810-28851232-R | CACACATGCACACCATTCAC |
| chr3:102027968-102028390-F | CCCACCAGCATTTCTGTACC |
| chr3:102027968-102028390-R | CTTATACCTCTGGCTGGCTCA |
| chr6:124893068-124893490-F | CCAGCTACTCCAGGGCCTA |
| chr6:124893068-124893490-R | TTTAGCACTGGATGCCCTG |

| **Supplementary Table S5: Primers used for RT-qPCR analysis.** | |
| --- | --- |
| **Primer Name** | **Primer Sequence (5'-3')** |
| M-WDR63-F | ACCGAGCCGCAGTATCTGA |
| M-WDR63-R | GCCGTCCTCTGTTCCAATGAG |
| H-WDR63-F | GGGCAGATTGTCATGTGGGA |
| H-WDR63-R | TATGGCCACGTTCCAACCTC |
| M-DNAH2-F | TGGATGTTAAGAACACCTCTTGG |
| M-DNAH2-R | GGTTCACCAGGGCTAACTCA |
| M-DNAH10-F | TGTGCCGAGTCATCAACCTA |
| M-DNAH10-R | AAAGGTCCTGGCAGATGTTG |
| NM_172864.3-F | ATGAACATGGAGAGCATGGGCC |
| NM_172864.3-R | CGTTTGTTCGTCTGTCATATCTTC |
| XM_017319579.3-F | ACGCGTCCTAGCAACAACTG |
| XM_017319579.3-R | AAGAAGGCAGCTTCTTGGCAG |
| M-GAPDH-F | AGGTCGGTGTGAACGGATTTG |
| M-GAPDH-R | GGGGTCGTTGATGGCAACA |
| H-GAPDH-F | GGAGCGAGATCCCTCCAAAAT |
| H-GAPDH-R | GGCTGTTGTCATACTTCTCATGG |

**Supplementary Movie S1: Sperm Motility in wild-type male mice.**

**Supplementary Movie S2: Sperm Motility in *Wdr63*-KO male mice.**

**Supplementary Data S1: The data of Mass spectrometric analysis.**
